# PDZD8 Disruption Causes Cognitive Impairment in Humans, Mice, and Fruit Flies

**DOI:** 10.1016/j.biopsych.2021.12.017

**Published:** 2022-08-15

**Authors:** Ahmed H. Al-Amri, Paul Armstrong, Mascia Amici, Clemence Ligneul, James Rouse, Mohammed E. El-Asrag, Andreea Pantiru, Valerie E. Vancollie, Hannah W.Y. Ng, Jennifer A. Ogbeta, Kirstie Goodchild, Jacob Ellegood, Christopher J. Lelliott, Jonathan G.L. Mullins, Amanda Bretman, Ruslan Al-Ali, Christian Beetz, Lihadh Al-Gazali, Aisha Al Shamsi, Jason P. Lerch, Jack R. Mellor, Abeer Al Sayegh, Manir Ali, Chris F. Inglehearn, Steven J. Clapcote

**Affiliations:** a School of Biomedical Sciences, University of Leeds, Leeds, United Kingdom; b School of Biology, University of Leeds, Leeds, United Kingdom; c Leeds Institute of Medical Research, University of Leeds, Leeds, United Kingdom; d School of Physiology, Pharmacology & Neuroscience, University of Bristol, Bristol, United Kingdom; e Wellcome Centre for Integrative Neuroimaging, Nuffield Department of Clinical Neuroscience, University of Oxford, Oxford, United Kingdom; f Wellcome Trust Sanger Institute, Hinxton, United Kingdom; g Institute of Life Science, Swansea University, Swansea, United Kingdom; h Department of Zoology, Faculty of Science, Benha University, Benha, Egypt; i National Genetic Centre, Royal Hospital, Muscat, Oman; j Genetics Department, Sultan Qaboos University Hospital, Muscat, Oman; k Mouse Imaging Centre, Hospital for Sick Children, Toronto, Ontario, Canada; l Centogene GmbH, Rostock, Germany; m Department of Paediatrics, College of Medicine & Health Sciences, United Arab Emirates University, Al Ain, United Arab Emirates; n Pediatrics Department, Tawam Hospital, Al Ain, United Arab Emirates; o Institute of Cancer and Genomic Sciences, University of Birmingham, Birmingham

**Keywords:** Brain structure, Endoplasmic reticulum, Intellectual disability, Long-term memory, PDZD8, Synaptic plasticity

## Abstract

**Background:**

The discovery of coding variants in genes that confer risk of intellectual disability (ID) is an important step toward understanding the pathophysiology of this common developmental disability.

**Methods:**

Homozygosity mapping, whole-exome sequencing, and cosegregation analyses were used to identify gene variants responsible for syndromic ID with autistic features in two independent consanguineous families from the Arabian Peninsula. For in vivo functional studies of the implicated gene’s function in cognition, *Drosophila melanogaster* and mice with targeted interference of the orthologous gene were used. Behavioral, electrophysiological, and structural magnetic resonance imaging analyses were conducted for phenotypic testing.

**Results:**

Homozygous premature termination codons in *PDZD8*, encoding an endoplasmic reticulum–anchored lipid transfer protein, showed cosegregation with syndromic ID in both families. *Drosophila melanogaster* with knockdown of the *PDZD8* ortholog exhibited impaired long-term courtship-based memory. Mice homozygous for a premature termination codon in *Pdzd8* exhibited brain structural, hippocampal spatial memory, and synaptic plasticity deficits.

**Conclusions:**

These data demonstrate the involvement of homozygous loss-of-function mutations in *PDZD8* in a neurodevelopmental cognitive disorder. Model organisms with manipulation of the orthologous gene replicate aspects of the human phenotype and suggest plausible pathophysiological mechanisms centered on disrupted brain development and synaptic function. These findings are thus consistent with accruing evidence that synaptic defects are a common denominator of ID and other neurodevelopmental conditions.

SEE VIDEO CONTENT ONLINE

Intellectual disability (ID) refers to a heterogeneous group of neurodevelopmental disorders affecting 2% to 3% of the general population, characterized by significant impairment in cognitive ability and adaptive behaviors. It is usually subdivided into nonsyndromic and syndromic forms, depending on the manifestation of additional physical, neurologic, and/or metabolic abnormalities. Typically identified in childhood because of delayed developmental milestones, affected individuals struggle with memory, problem solving, language, and visual comprehension, reflected by an IQ score of <70 (1).

ID has high phenotypic variability and etiologic diversity. Based on the IQ score, ID can be classified as mild (50–69), moderate (35–49), severe (20–34), or profound (under 20) (2). Among the known causes, approximately 50% of ID cases have an early environmental etiology, such as intrauterine exposure to alcohol, the most common nonheritable cause of ID (3). The remaining ∼50% of ID cases of known cause have a genetic etiology, such as chromosomal abnormalities or mutations in specific genes (4).

Because ID negatively affects fecundity, dominant autosomal variants occurring de novo may contribute to a large proportion of sporadic cases, particularly in outbred Western populations (5). Autosomal recessive variants play a significant role in ID in populations with frequent parental consanguinity, such as in the Middle East (6,7). Defects in more than 700 genes have been implicated in ID, and a significant overlap has been noted with genes identified in other neurodevelopmental disorders such as autism spectrum disorder (ASD) (8). Functional categorization of the encoded proteins has revealed significant enrichment of proteins involved in glutamatergic synapse structure and function (9, 10, 11). Despite the considerable progress in understanding, no treatment is currently available for ID, and at least 50% of the estimated genetic causes of ID remain unknown (12).

Herein, we report the clinical features and molecular diagnosis of two independent consanguineous families affected by syndromic ID from the Arabian Peninsula. Through the application of homozygosity mapping and whole-exome sequencing (WES), we report that all affected individuals are homozygous for premature termination codons (PTCs) in *PDZD8* (formerly *PDZK8*), a gene of five exons located at 10q25.3-q26.11, encoding a 1154 aa endoplasmic reticulum (ER) transmembrane (TM) protein.

In neurons, depletion of PDZD8 has been shown to impair endosomal homeostasis (13), decrease the proximity of the ER and mitochondria (14), and decrease calcium ion (Ca^2+^) uptake by mitochondria following synaptic transmission–induced Ca^2+^ release from the ER (15).

Because assessing human gene function in cognition is challenging, we used a cross-species approach. We report that targeted interference of the *PDZD8* orthologs in fruit flies and mice leads to long-term memory, brain structural, and synaptic plasticity deficits. Our findings are consistent with accruing evidence that glutamatergic synapse dysfunction represents a common underlying pathogenic mechanism in ID and other neurodevelopmental disorders (8, 9, 10).

## Methods and Materials

For more detailed methodology, see the Supplemental Experimental Procedures.

### Ethical Approvals

The human study was approved by the Sultan Qaboos University Ethical Committee. Informed consent was obtained from the parents of the affected individuals using a process that adhered to the tenets of the Declaration of Helsinki. The mouse study was conducted in accordance with the UK Animals (Scientific Procedures) Act 1986 under UK Home Office licenses and approved by institutional Animal Welfare and Ethical Review Bodies.

### Sequencing and Variant Identification

Homozygosity mapping and WES were conducted as described previously (16). Segregation in families was confirmed by polymerase chain reaction and Sanger sequencing.

### *Drosophila melanogaster*

The UAS-*CG10362*-RNAi (v.8317; UAS-RNAi) line (17) was crossed with the *Act5C*-Gal4 ubiquitous driver line to induce ubiquitous expression of a specific 326-bp hairpin structure (*CG10362*-RNAi) that inhibits expression of the target *CG10362* gene via RNA interference. Behavioral testing was performed as described previously (18, 19, 20).

### Mice

C57BL/6NTac-*Pdzd8*^tm1b(EUCOMM)Wtsi^ mice were generated by replacing an 835-bp sequence including exon 3 with a *lacZ* expression cassette, which created a frameshift that changed the phenylalanine (F) and isoleucine (I) at positions 333 and 334 to an asparagine (N) and a termination codon (∗) (p.F333Nfs1∗) (Figure S1A, C) (21). Heterozygotes were intercrossed to generate *Pdzd8* homozygous mutant (*Pdzd8*^*tm1b*^; tm1b) and heterozygous and wild-type (WT) littermates for phenotypic testing.

### Mouse Behavioral Testing

Behavioral testing of early adults over 8 weeks of age was performed as described previously (22, 23, 24, 25).

### Electrophysiology

Extracellular field recordings using transverse hippocampal slices prepared from 4- to 6-week-old mice were performed as described previously (26). Three different long-term potentiation (LTP) induction protocols were used. Theta burst stimulation (TBS) consisted of 10 bursts at 5 Hz, where each burst consisted of five stimuli at 100 Hz. This was applied either once (1× TBS) or three times separated by 10 seconds (3× TBS). High-frequency stimulation (HFS) consisted of one burst of 100 stimuli at 100 Hz (1× HFS).

### Structural Magnetic Resonance Imaging

For high-resolution structural magnetic resonance imaging, 16-week-old mice were terminally anesthetized and intracardially perfused. Samples were processed, imaged, and analyzed as described previously (27). A linear model with genotype and sex as predictors was fitted to the absolute (mm^3^) and relative volume of every region independently and to every voxel independently in the brains of *Pdzd8*^*tm1b*^ and WT mice, with a false discovery rate threshold of 1%.

## Results

### Clinical Features

**Figure 1:**
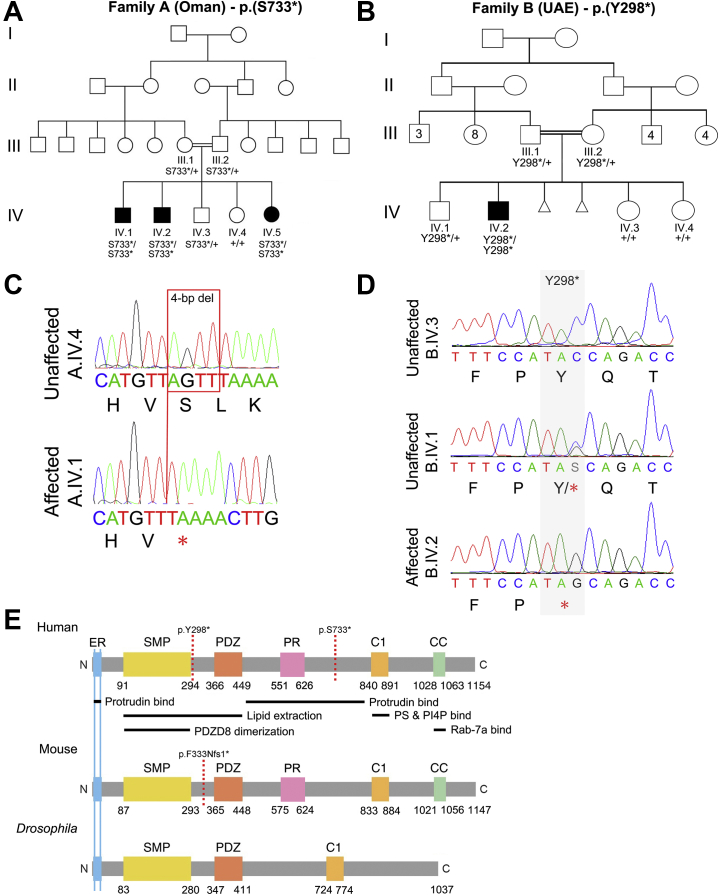
Two families with *PDZD8* mutations. **(A)** Pedigree of four-generation family A showing cosegregation of *PDZD8* p.(S733∗) homozygosity with syndromic ID in 3 affected siblings (represented by filled symbols). **(B)** Pedigree of four-generation family B showing cosegregation of *PDZD8* p.(Y298∗) homozygosity with syndromic ID in the affected individual (represented by filled symbol). Two progeny who died in utero are represented by small triangles. The numbers in generation III indicate brothers and sisters of the parents (B.III.1 and B.III.2). **(C)** Sanger sequence chromatograms showing the *PDZD8* 4-bp (AGTT) deletion (c.2197_2200del) identified in family A. **(D)** Sanger sequence chromatograms showing the *PDZD8* nonsense mutation (c.894C>G) identified in family B. **(E)** Schematic diagram depicting domain structure and functions of PDZD8 in human (Q8NEN9; top), mouse (B9EJ80; middle), and *Drosophila* (Q9VYR9; bottom). The ER-TM domain (2–24 aa) and a region between the PDZ and phorbol-ester/diacylglycerol binding (C1) domains (466–797 aa) are required for interaction with protrudin (13,34,49). The SMP domain is required for the formation of PDZD8 dimers or oligomers (49). The SMP and PDZ domains are required for the extraction of lipids from the ER to late endosomes and lysosomes (13,49). The C1 domain is required for interaction with PS and PI4P associated with the late endosome/lysosome membrane (13,50). The CC domain is required for interaction with Rab-7a (34,49,51). Black horizontal lines indicate interactor binding sites; broken vertical red lines indicate the location of PTC (human: p.Y298∗ & p.S733∗; mouse: p.F333Nfs1∗). Numbering is from published sources (13,34,35). C, carboxyl-terminus; CC, coiled-coil; ER, endoplasmic reticulum; ID, intellectual disability; N, amino-terminus; PR, proline-rich; PS, phosphatidylserine; PI4P, phosphatidylinositol 4-phosphate; PDZ, PSD-95/DlgA/ZO-1-like; PTC, premature termination codon; SMP, synaptotagmin-like mitochondrial lipid-binding; TM, transmembrane; UAE, United Arab Emirates.

Family A consists of 3 affected (A.IV.1, A.IV.2, and A.IV.5) and 2 unaffected (A.IV.3 and A.IV.4) siblings born to consanguineous parents (first cousins) (A.III.1 and A.III.2) within an extended Omani pedigree (Figure 1A). Clinical examination revealed that all affected individuals have moderate to severe ID with autistic features, myopathy, and facial dysmorphism (myopathic face with orbital hypertelorism, malar flattening, open mouth, and high-arched palate). In addition, each affected sibling had other specific health problems, as detailed in Table 1. Both the father (A.III.2) and an unaffected male sibling (A.IV.3) had mild autistic features, and both parents (A.III.1 and A.III.2) had mild myopathy and reduced facial expression.

**Table 1:** Clinical Features of Patients With Mutations in *PDZD8*

Characteristic	Family A Affected Individuals			Family B Affected Individual
	A.IV.1	A.IV.2	A.IV.5	B.IV.2
Consanguinity	Yes	Yes	Yes	Yes
Ethnic Origin	Omani	Omani	Omani	Emirati
Genotype, Mat/Pat	p.(S733∗)/p.(S733∗); c.2197_2200del/c.2197_2200del	p.(S733∗)/p.(S733∗); c.2197_2200del/c.2197_2200del	p.(S733∗)/p.(S733∗); c.2197_2200del/c.2197_2200del	p.(Y298∗)/p.(Y298∗); c.894C>G/c.894C>G
Sex	Male	Male	Female	Male
Age, Years	30	25	17	7
Developmental Delay	Yes	Yes	Yes	Yes
Intellectual Disability	Yes (severe)	Yes (moderate)	Yes (severe)	Yes (moderate)
Autistic Features	Yes	Yes (mild)	Yes	Yes (mild)
Facial Dysmorphism	Yes	Yes	Yes	Yes
Orbital Hypertelorism	Yes	Yes	Yes	Yes
Myopia	No	No	Yes	Yes
Myopathy	Yes	Yes (mild)	Yes	No
Epilepsy	No	Yes (controlled)	Yes	No
Congenital Heart Defects	No	No	Yes	No
Marfanoid Habitus	Yes	No	Yes	No
Other Behavioral Problems	No	Yes (OCD)	Yes (ADHD; insomnia)	Yes (ADHD)
Brain Scan Findings	ND	ND	Hypoplasia of splenium of corpus callosum	Normal
Other Findings	No	No	Amblyopia, cleft palate, scoliosis	Bilateral ptosis, astigmatism, overlapping toes

Nucleotide and residue numbering are based on NM_173791.5.

ADHD, attention-deficit/hyperactivity disorder; Mat, maternal; ND, not determined; OCD, obsessive-compulsive disorder; Pat, paternal.

Family B consists of 1 affected (B.IV.2) and 3 unaffected (B.IV.1, B.IV.3, and B.IV.4) siblings born to first cousin parents (B.III.1 and B.III.2) within an Emirati pedigree (Figure 1B). On clinical examination at 4 years of age, the affected male (B.IV.2) presented with delayed speech, moderate ID, mild autistic features (echolalia, jumping, hand flapping, lack of eye contact), attention deficit, dysmorphic features (low-set ears with simple helix, bilateral ptosis), and other specific health problems detailed in Table 1.

### Identification of *PDZD8* Mutations

The pedigree structures of families A and B suggested autosomal recessive transmission of a homozygous mutant allele from a common ancestor as the most likely explanation for syndromic ID in each family. In family A, homozygosity mapping using single nucleotide polymorphism array data in affected males A.IV.1 and A.IV.2 and variants extracted from WES in affected female A.IV.5 identified homozygous regions on chromosomes 6 (2.57 Mb), 10 (28.28 Mb), 13 (5.20 Mb), and 17 (8.38 Mb) shared by all three (Figure S2A).

WES in subject A.IV.5 revealed 9032 variants in these shared homozygous regions. After filtering for rare variants predicted to be pathogenic followed by segregation analysis, only variants in *ANKRD2* [NM_020349.4: c.982C>T; p.(R328W)] and *PDZD8* [NM_173791.5: c.2197_2200del; p.(S733∗)] remained, both within the 28.28-Mb region on chromosome 10 (Figure 1A and Figure S2B).

The *PDZD8* c.2197_2200del variant deletes four base pairs in exon 5, introducing a frameshift and PTC (Figure 1C and Table 2) absent from gnomAD. *ANKRD2* variant c.982C>T lies in exon 9, is present at a frequency of 0.00001961 with no homozygotes in gnomAD version 2.1.1 (control subjects) (28), and causes missense change p.(R328W) (Figure S2C), predicted by PolyPhen-2 to be possibly damaging and by SIFT as deleterious (29,30) (Table S1). In a structural model of ANKRD2, the p.(R328W) variant appears to change the general conformation of the protein (Figure S2E). Both variants were absent from 50 ethnically matched Omani control DNAs.

**Table 2:** Mutations Identified in *PDZD8*

Family	Ethnicity	Genotype	*PDZD8* Modification	Nucleotide Change	Frequency in gnomAD	CADD Score
A	Omani	Homozygous	p.(S733∗)	c.2197_2200del	0	35
B	Emirati	Homozygous	p.(Y298∗)	c.894C>G	0	37

Nucleotide and residue numbering are based on NM_173791.5.

CADD, Combined Annotation Dependent Depletion.

Because very little ANKRD2 (UniProtKB: Q9GZV1) is detected in the human brain (31) and primary immunodeficiency is caused by missense changes in the gene (32), the p.(R328W) variant appears unlikely to be responsible for ID in family A. However, because *ANKRD2* is upregulated in congenital myopathies (33), p.(R328W) homozygosity may contribute to myopathy in family A.

In family B, WES of the affected sibling (B.IV.2), with filtering for predicted pathogenic variants and segregation analysis, revealed a homozygous nonsense variant in *PDZD8* exon 2 [NM_173791: c.894C>G; p.(Y298∗)] (Figure 1D and Table 2) as the most likely cause of his condition. The p.(Y298∗) variant is absent from gnomAD, and no other variants that potentially explain the phenotype were identified in his exome. gnomAD control datasets list five other predicted loss-of-function variants in *PDZD8*, none homozygous, and constraint metrics indicate that *PDZD8* is extremely intolerant to loss-of-function variation (28).

*PDZD8* encodes an integral ER protein (UniProtKB: Q8NEN9) anchored to the membrane by an N-terminal TM helical domain (2–24 aa), which is followed by a synaptotagmin-like mitochondrial lipid-binding domain (91–294 aa), a PDZ (PSD-95/DlgA/ZO-1-like) domain (366–449 aa), a proline-rich region (551–626 aa), a C1 (phorbol-ester/diacylglycerol binding) domain (840–891 aa), and a coiled-coil domain (1028–1063 aa) (13,34,35). If the p.(Y298∗) and p.(S733∗) variants evade nonsense-mediated messenger RNA (mRNA) decay (NMD) (36), truncated PDZD8 proteins lacking 857 (p.Y298∗) or 422 (p.S733∗) C-terminal amino acids would be produced (Figure 1E and Figure S3). *PDZD8* is highly expressed throughout the human brain (31), including all subclasses of GABAergic (gamma-aminobutyric acidergic) and glutamatergic neurons in the adult primary motor cortex (37) (Figure S4). Our analysis of bulk RNA sequencing data from the BrainSpan project (38) revealed that *PDZD8* expression is relatively stable from 8 weeks after conception to early adulthood (23 years) across regions of the developing human brain (Figure S5), suggesting a role in neurodevelopment, making *PDZD8* a strong candidate for involvement in ID.

### Long-term Memory Deficit in *Drosophila* Knockdown Model

**Figure 2:**
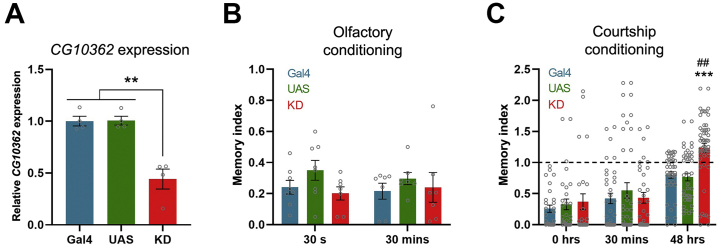
Associative learning and memory in *CG10362* KD flies. **(A)** Expression of *CG10362* in four pools of 8 to 10 whole adult *CG10362*-KD (KD: 0.4417 ± 0.09643), UAS-RNAi (UAS: 1.006 ± 0.04253), and *Act5C*-Gal4 (Gal4: 1.001 ± 0.04712) male flies (one-way analysis of variance: *F*_2,9_ = 23.68, *p* < .0001; post hoc Bonferroni’s correction, Gal4 vs. UAS: *p* = 1.0, KD vs. UAS: *p* = .001, KD vs. Gal4: *p* = .001). **(B)** Aversive olfactory conditioning assay memory indices 30 seconds (learning) after training of KD (*n* = 8; 0.2393 ± 0.0442), UAS (*n* = 8; 0.35 ± 0.0634), and Gal4 (*n* = 7; 0.2024 ± 0.043) flies (one-way analysis of variance: *F*_2,20_ = 2.189, *p* = .1382) and 30 minutes (short-term memory) after training of KD (*n* = 7; 0.2405 ± 0.0956), UAS (*n* = 7; 0.2969 ± 0.0394), and Gal4 (*n* = 7; 0.2161 ± 0.0512) flies (Kruskal-Wallis: χ^2^_2_ = 1.2764, *p* = .5282). **(C)** Courtship conditioning assay memory indices immediately (0 hours) after training of KD (*n* = 17; 0.4327 ± 0.1782), UAS (*n* = 20; 0.3493 ± 0.0994), and Gal4 (*n* = 18; 0.3169 ± 0.0772) flies (Kruskal-Wallis: χ^2^_2_ = 0.8324, *p* = .6595); 30 minutes after training of KD (*n* = 22; 0.4868 ± 0.1085), UAS (*n* = 24; 0.5326 ± 0.1542), and Gal4 (*n* = 19; 0.5144 ± 0.1067) flies (Kruskal-Wallis: χ^2^_2_ = 0.8672, *p* = .6482); and 48 hours after training of KD (*n* = 33; 1.2102 ± 0.0902), UAS (*n* = 27; 0.7301 ± 0.0786), and Gal4 (*n* = 25; 0.8123 ± 0.0669) flies (one-way analysis of variance: *F*_2,82_ = 10.52, *p* < .0001; post hoc Bonferroni’s correction, Gal4 vs. UAS: *p* = 1.0, KD vs. UAS: *p* = < .001, KD vs. Gal4: *p* = .003). Above dotted line (1.0) indicates no memory. Data are plotted as mean ± SEM. ∗∗*p* < .01 vs. controls; ^##^*p* < .01 vs. Gal4; ∗∗∗*p* < .001 vs. UAS. KD, knockdown; UAS, upstream activation sequence.

To assess PDZD8 function in cognition, we targeted the orthologous gene in *D. melanogaster*. The *PDZD8* ortholog, *CG10362* (FlyBase ID: FBgn0030358), encodes a 1037 aa protein (LD34222p; NP_572771.1; UniProtKB: Q9VYR9) that has a similar domain structure but relatively low amino acid conservation compared with human PDZD8 (24% identity). *Drosophila* PDZD8 has an N-terminal TM domain followed by a synaptotagmin-like mitochondrial lipid-binding domain, a PDZ domain, and a C1 domain but, unlike mammalian PDZD8, lacks a C-terminal coiled-coil domain (Figure 1E) (39). In adult flies, *CG10362* expression is enriched in head, eye, brain, and thoracico-abdominal ganglion (noncephalic central nervous system) tissue (40). RNA interference–mediated knockdown (KD) of *CG10362* reduced the level of the target *CG10362* transcript by 56.32 ± 9.20% in adult *Act5C*-Gal4 x UAS-RNAi F_1_ (*CG10362*-KD) males upon comparison with UAS-RNAi and *Act5C*-Gal4 control animals (Figure 2A).

In the aversive olfactory conditioning assay, the ability of *CG10362*-KD flies to memorize a novel association between an odor and mechanical shock for 30 seconds or 30 minutes was unaltered (Figure 2B). In the courtship conditioning assay, *CG10362*-KD males demonstrated reduced suppression of courtship behavior 48 hours, but not immediately or 30 minutes, after exposure to a premated female (Figure 2C). Unconditioned *CG10362*-KD males displayed robust courting behavior toward virgin females (data not shown). These results suggest that KD of *CG10362* impairs long-term courtship-based memory but does not affect learning, short-term memory, or basal sexual behavior.

### *Pdzd8*^*tm1b*^ Mice Exhibit Restricted Growth and Brain Structural Alterations

To gain further insight to the cognitive effects of PDZD8 disruption, we studied the pre-existing *Pdzd8*^*tm1b*^ mouse line, generated in the EUCOMM (European Conditional Mouse Mutagenesis) program (21), which harbors the mutation F333Nfs1∗ that closely models the PTCs identified in families A and B (Figure 1E). The mouse ortholog, *Pdzd8*, encodes a 1147 aa protein (UniProtKB: B9EJ80) that has 87% aa conservation with, and a similar domain structure to, human PDZD8. Real-time quantitative reverse transcription polymerase chain reaction confirmed the absence of *Pdzd8* mRNA including exon 3 in *Pdzd8*^*tm1b*^ mouse brain (Figure S6). Western blotting using an antibody to PDZD8, with an epitope between the C-terminal C1 and coiled-coil domains (Figure S3), detected a ∼140-kDa protein in WT mouse brain, which was 63.24 ± 3.84% less abundant in heterozygous samples and absent from *Pdzd8*^*tm1b*^ samples (Figure S7), confirming the loss of full-length PDZD8 in *Pdzd8*^*tm1b*^ mice.

**Figure 3:**
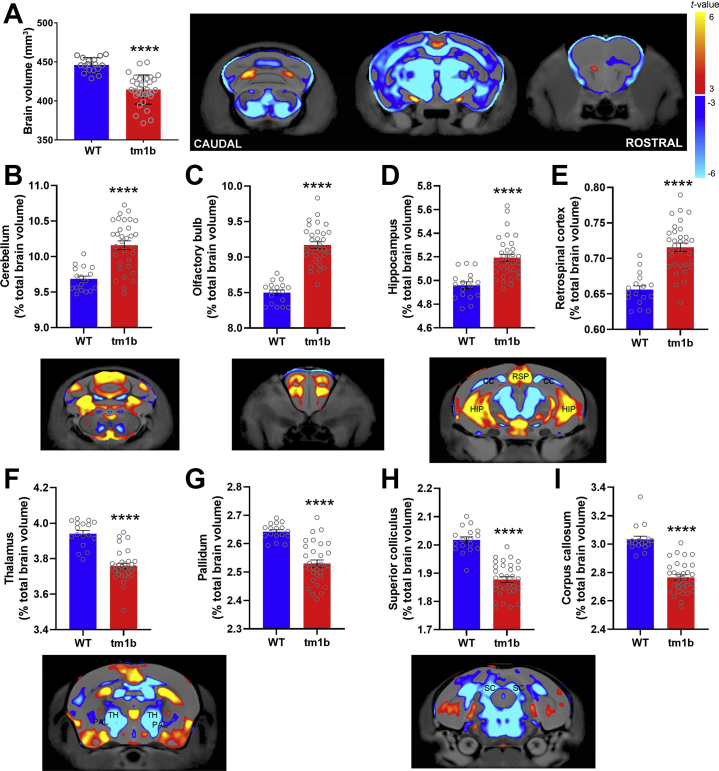
Voxelwise volumetric differences in whole brain and specific brain regions in *Pdzd8*^*tm1b*^ mice determined by high-resolution structural magnetic resonance imaging. Significant differences in volume between *Pdzd8*^*tm1b*^ (*n* = 32 [10 males, 22 females]) and WT (*n* = 17 [7 males, 10 females]) are indicated by red (increased volume) and blue (reduced volume) contour shading on two-dimensional coronal slice images of the brain. **(A)** Absolute brain volume (mm^3^). **(B)** Cerebellum: relative volume (% total brain volume). **(C)** Olfactory bulb: relative volume (% total brain volume). **(D)** Hippocampus: relative volume (% total brain volume). **(E)** Retrosplenial cortex: relative volume (% total brain volume). **(F)** Thalamus: relative volume (% total brain volume). **(G)** Pallidum: relative volume (% total brain volume). **(H)** Superior colliculus: relative volume (% total brain volume). **(I)** Corpus callosum: relative volume (% total brain volume). ∗∗∗∗*p* < .0001 vs. WT. WT, wild-type.

When intercrossing heterozygotes, *Pdzd8*^*tm1b*^ pups were weaned at rates 36% and 48% lower than the expected Mendelian genotypic ratio in two separate colonies. Surviving *Pdzd8*^*tm1b*^ mice appeared healthy but had a lower body weight and growth rate between 4 and 16 weeks of age (Figure S8A–D). Soft tissue mass (Figure S8E, F) and body length (Figure S8G, H) were reduced in 14-week-old *Pdzd8*^*tm1b*^ mice. Structural magnetic resonance imaging to identify changes in brain morphology revealed a 7.07 ± 0.74% decrease in total brain volume in 16-week-old *Pdzd8*^*tm1b*^ mice compared with WT littermates (Figure 3A). To assess differences in specific brain regions, the volume of each region was normalized to absolute brain volume. *Pdzd8*^*tm1b*^ mice showed an increased relative volume of the cerebellum, olfactory bulb, hippocampus, and retrosplenial cortex (Figure 3B–E) but a decreased relative volume of the thalamus, pallidum, superior colliculus, and corpus callosum (Figure 3F–I), indicative of brain structural alterations. Representative structural images for *Pdzd8*^*tm1b*^ and WT mice of each sex are shown in Figure S9.

### *Pdzd8*^*tm1b*^ Mice Exhibit Spontaneous Stereotypies and Decreased Anxiety-like Behavior

In the home cage, adult *Pdzd8*^*tm1b*^ mice frequently showed spontaneous repetitive hindlimb jumping behavior (Figure S10A), typically preceded by rearing against the wall, when housed in a mixed-genotype group (Video S1) or alone (Video S2). This stereotyped motor behavior was not observed in WT littermates.

**Figure 4:**
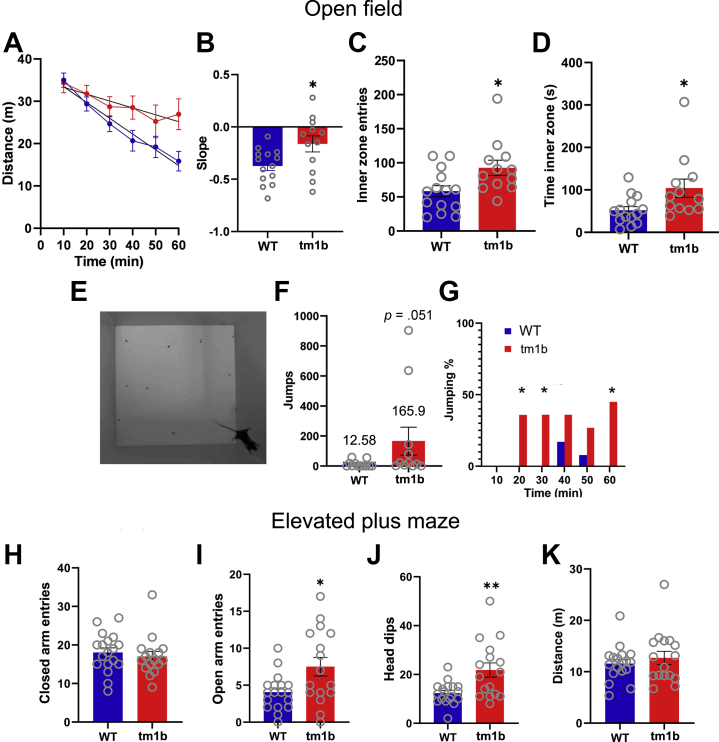
Behavioral differences of *Pdzd8*^*tm1b*^ mice in OF and EPM. **(A)** Distance traveled (m) by *Pdzd8*^*tm1b*^ (*n* = 12) and WT (*n* = 14) mice in 10-minute intervals in OF, with lines of best fit shown (two-way repeated-measures analysis of variance, genotype: *F*_1,24_ = 3.037, *p* = .094; time: *F*_2.44,58.65_ = 23.17, *p* < .0001; interaction: *F*_2.44,58.65_ = 3.795, *p* = .021). **(B)** Slope of habituation curve of *Pdzd8*^*tm1b*^ (−0.1625 ± 0.07680) and WT (−0.3727 ± 0.04413) mice (independent *t* test: *t*_24_ = 2.458, *p* = .028). **(C)** Number of entries by *Pdzd8*^*tm1b*^ (92.67 ± 11.28) and WT (58.36 ± 8.195) mice to OF inner zone (independent *t* test: *t*_24_ = −2.508, *p* = .019). **(D)** Time (s) spent by *Pdzd8*^*tm1b*^ (104.00 ± 21.44) and WT (51.69 ± 8.965) mice in OF inner zone (independent *t* test: *t*_24_ = −2.710, *p* = .012). **(E)** Representative image of hindlimb jumping by *Pdzd8*^*tm1b*^ mouse in OF. **(F)** Number of jumps by *Pdzd8*^*tm1b*^ (*n* = 11; 165.9 ± 92.78) and WT (*n* = 12; 12.58 ± 6.289) mice in OF (Mann-Whitney *U* test: *U* = 97.00, *p* = .051). (**G**) Percentage of *Pdzd8*^*tm1b*^ and WT mice making more than 10 jumps per 10-minute interval in OF (Fisher’s exact test, 10–20 min: *p* = .037; 20–30 min: *p* = .037; 50–60 min: *p* = .037). **(H)** Number of entries to closed arms by *Pdzd8*^*tm1b*^ (17.0 ± 1.317) and WT (18.06 ± 1.184) mice in EPM (independent *t* test: *t*_32_ = 0.597, *p* = .5541). (**I**) Number of entries to open arms by *Pdzd8*^*tm1b*^ (7.5 ± 1.258) and WT (4.056 ± 0.5686) mice in EPM (Welch’s *t* test: *t*_32_ = −2.494, *p* = .021). **(J)** Number of head dips by *Pdzd8*^*tm1b*^ (21.81 ± 2.91) and WT (12.33 ± 1.06) mice in EPM (Welch’s *t* test: *t*_32_ = −3.061, *p* = .006). **(K)** Total distance traveled (m) by *Pdzd8*^*tm1b*^ (*n* = 16; 12.69 ± 1.27) and WT (*n* = 18; 11.60 ± 0.79) mice in EPM (Mann-Whitney *U* test: *U* = 133, *p* = .720). Data are plotted as mean ± SEM. ∗*p* < .05; ∗∗*p* < .01 vs. WT. EPM, elevated plus maze; OF, open field; WT, wild-type.

To assess *Pdzd8*^*tm1b*^ mouse behavior in more detail, we observed mice in the open field test. In the novel arena, the total distances traveled were comparable between *Pdzd8*^*tm1b*^ and WT littermates (Figure S10B), but the ambulatory activity of *Pdzd8*^*tm1b*^ mice decreased more slowly over the course of the 1-hour test (Figure 4A, B), indicative of reduced habituation (41, 42). *Pdzd8*^*tm1b*^ mice had more entries to (Figure 4C), and spent more time (Figure 4D) and traveled further in (Figure S10C), the center of the arena (Figure S10D), suggestive of less anxiety-like behavior. *Pdzd8*^*tm1b*^ mice also exhibited repetitive jumping behavior in the open field (Figure 4E–G).

In the elevated plus maze, *Pdzd8*^*tm1b*^ mice exhibited a greater frequency of open arm entries (Figure 4I) and head dips (protruding the head over the edge of an open arm and down toward the floor) (Figure 4J) than WT littermates, suggesting decreased anxiety and increased exploration (43, 44, 45).

### Long-term Spatial Memory and TBS-Induced LTP Are Impaired in *Pdzd8*^*tm1b*^ Mice

**Figure 5:**
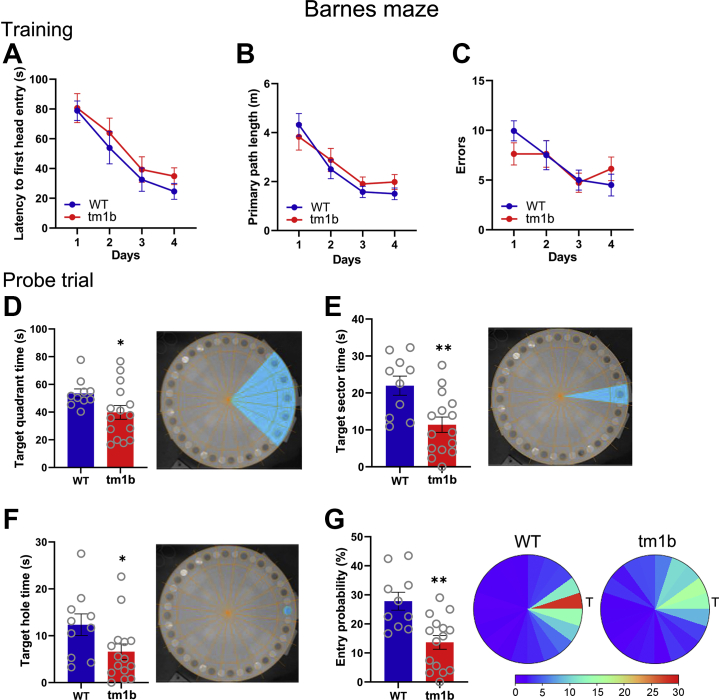
Performance of *Pdzd8*^*tm1b*^ (*n* = 15) and WT (*n* = 10) mice in Barnes maze. **(A)** Latency (s) to first head entry to escape hole (Friedman’s analysis of variance, *Pdzd8*^*tm1b*^: χ^2^_3_ = 19.88, *p* < .0001; WT: χ^2^_3_ = 21.30, *p* < .0001. Mann-Whitney *U* test, day 1: *U* = 79.50, *p* = .80; day 2: *U* = 85.50, *p* = .56; day 3: *U* = 78.00, *p* = .868; day 4: *U* = 98.00, *p* = .216). **(B)** Primary path length (m) (Friedman’s analysis of variance, *Pdzd8*^*tm1b*^: χ^2^_3_ = 11.32, *p* = .01; WT: χ^2^_3_ = 23.88, *p* < .0001. Mann-Whitney *U* test, day 1: *U* = 60.00, *p* = .42; day 2: *U* = 76.00, *p* = 1.00; day 3: *U* = 83.00, *p* = .68; day 4: *U* = 92.00, *p* = .367). **(C)** Number of errors. (Friedman’s analysis of variance, *Pdzd8*^*tm1b*^: χ^2^_3_ = 10.50, *p* = .015; WT: χ^2^_3_ = 9.39, *p* = .024. Mann-Whitney *U* test, day 1: *U* = 48.50, *p* = .144; day 2: *U* = 72.00, *p* = .892; day 3: *U* = 67.50, *p* = .683; day 4: *U* = 94.00, *p* = .311). (**D)** Time (s) spent by *Pdzd8*^*tm1b*^ (39.76 ± 4.983) and WT (53.45 ± 3.273) mice in target quadrant (Welch’s *t* test: *t*_22.24_ = 2.296, *p* = .031. One-sample *t* test, *Pdzd8*^*tm1b*^ vs. chance [20]: *t*_14_ = 3.965, *p* = .0014; WT vs. chance [20]: *t*_9_ = 10.22, *p* < .0001). **(E)** Time (s) spent by *Pdzd8*^*tm1b*^ (11.39 ± 2.088) and WT (21.96 ± 2.579) mice in target sector (independent *t* test: *t*_23_ = 3.223, *p* = .004. One-sample *t* test, *Pdzd8*^*tm1b*^ vs. chance [4]: *t*_14_ = 3.540, *p* = .0033; WT vs. chance [4]: *t*_9_ = 6.965, *p* < .0001). **(F)** Time (s) spent by *Pdzd8*^*tm1b*^ (6.587 ± 1.643) and WT (12.35 ± 2.3) mice within target hole annulus (Mann-Whitney *U* test: *U* = 36.00, *p* = .031). **(G)** Left, entry probability (%) of *Pdzd8*^*tm1b*^ (13.64 ± 2.359) and WT (27.8 ± 3.086) mice into the target hole annulus (independent *t* test: *t*_23_ = 3.69, *p* = .001). Right, heat maps of mean entry probability (%) of *Pdzd8*^*tm1b*^ (right) and WT (left) mice. Data are plotted as mean ± SEM. ∗*p* < .05; ∗∗*p* < .01 vs. WT. T, target sector; WT, wild-type.

In the Y maze, *Pdzd8*^*tm1b*^ and WT mice showed roughly equivalent levels of spontaneous alternation, a measure of spatial working memory (Figure S11D). In the Barnes maze, the performance levels of *Pdzd8*^*tm1b*^ and WT mice were similar over the 4 days of training (Figure 5A–C and Figure S11E, F), demonstrating intact spatial learning. During the probe trial 24 hours after the last training trial, *Pdzd8*^*tm1b*^ mice spent less time in the target quadrant, target sector, and target hole annulus and had a lower probability of entering the target hole than WT mice (Figure 5D–G), indicative of a hippocampal-dependent spatial memory impairment in *Pdzd8*^*tm1b*^ mice.

**Figure 6:**
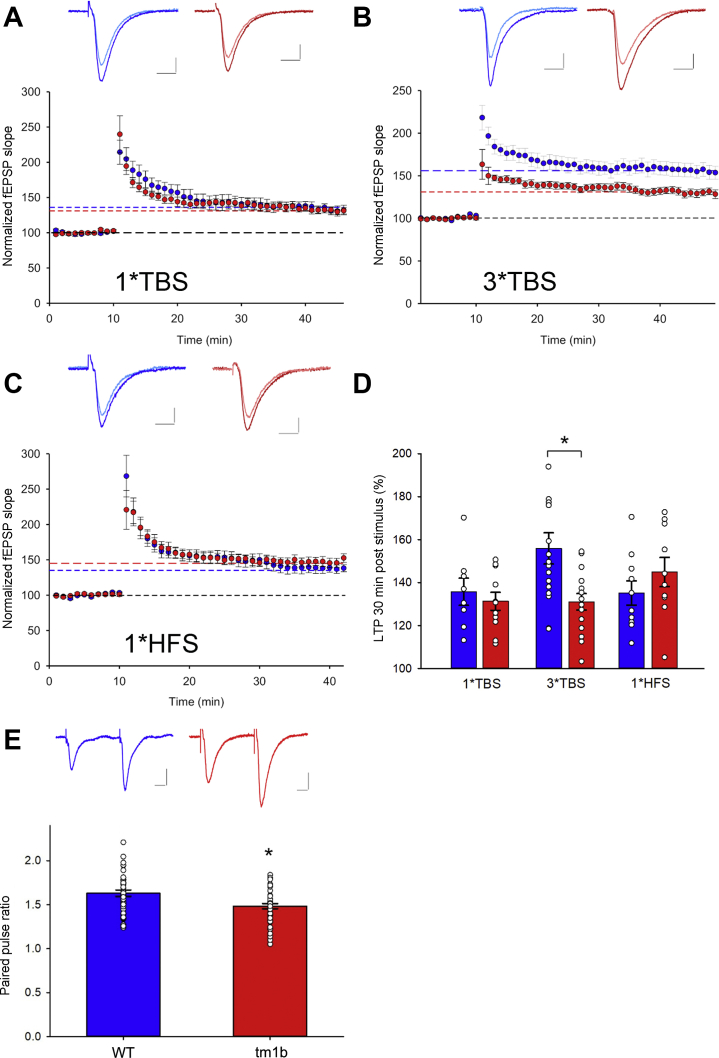
Analysis of hippocampal (long-term potentiation) in *Pdzd8*^*tm1b*^ mice. **(A)** Normalized change in fEPSP (% baseline) induced by 1× TBS in *Pdzd8*^*tm1b*^ (131 ± 4%; *n* = 11) and WT (136 ± 6%; *n* = 8) mice. **(B)** Normalized change in fEPSP (% baseline) induced by 3× TBS in *Pdzd8*^*tm1b*^ (131 ± 4%; *n* = 15) and WT (156 ± 7%; *n* = 16) mice. Scale bars = 0.3 mV and 10 ms in **(A)** and (**B**). **(C)** Normalized change in fEPSP (% baseline) induced by 1× HFS in *Pdzd8*^*tm1b*^ (145 ± 7%; *n* = 10) and WT (135 ± 6%; *n* = 12) mice. Insets: representative traces before (WT, light blue; *Pdzd8*^*tm1b*^, pink) and after (WT, blue; *Pdzd8*^*tm1b*^, red) the induction protocol. Scale bars = 0.2 mV and 10 ms. **(D)** Facilitation of fEPSP (% baseline) at 30 minutes after 1× TBS, 3× TBS, and 1× HFS. **(E)** Paired-pulse ratio of *Pdzd8*^*tm1b*^ (1.48 ± 0.03; *n* = 43) and WT (1.63 ± 0.04; *n* = 39) mice with 50-ms stimulus interval. Representative traces of WT (blue) and *Pdzd8*^*tm1b*^ (red) slices. Scale bars = 0.2 mV and 100 ms. Data are plotted as mean ± SEM. ∗*p* < .05 vs. WT. fEPSP, field excitatory postsynaptic potential; HFS, high-frequency stimulation; TBS, theta burst stimulation; WT, wild-type.

We next used electrophysiology to examine synaptic plasticity in acute hippocampal slices from *Pdzd8*^*tm1b*^ mice. Experimentally induced LTP of synaptic transmission is a widely accepted model of synaptic plasticity that involves molecular and cellular processes engaged during the biological consolidation of memories (46). Three different stimulation protocols to induce LTP at Schaffer collateral–CA1 stratum radiatum synapses were used: 3× TBS (a maximal stimulation that induces saturated LTP), 1× TBS, and 1× HFS (submaximal). The magnitudes of LTP evoked by 1× TBS and 1× HFS at 30 minutes after stimulation were comparable between *Pdzd8*^*tm1b*^ and WT slices (Figure 6A, C). However, the LTP evoked by 3× TBS was diminished in *Pdzd8*^*tm1b*^ compared with WT slices (Figure 6B), indicating that *Pdzd8*^*tm1b*^ mice have a specific deficit in 3× TBS-evoked LTP and are not capable of generating as much synaptic potentiation as WT mice.

To evaluate presynaptic short-term plasticity at Schaffer collateral–CA1 synapses in *Pdzd8*^*tm1b*^ and WT slices, we used paired-pulse stimulation with a 50-ms interval between the first and second pulses. Both genotypes exhibited paired-pulse facilitation of excitatory synaptic transmission (Figure 6E), postulated to result from a transient increase in Ca^2+^ levels in the presynaptic terminal. However, the lower paired-pulse facilitation of *Pdzd8*^*tm1b*^ slices suggests a higher initial probability of neurotransmitter release associated with the first pulse or reduced residual Ca^2+^ resulting from altered Ca^2+^ uptake (47).

## Discussion

We have identified homozygous PTC variants p.(Y298∗) and p.(S733∗) in *PDZD8* cosegregating with syndromic ID in two independent consanguineous families from the Arabian Peninsula. Such mutations are by default often considered loss-of-function events for the protein-coding genes that harbor them, in part because of the assumption that the PTC-containing mRNA is degraded by NMD (36). Because NMD is less efficient for PTCs in the last exon (48), it is possible that p.(S733∗) located in the last exon of *PDZD8* may evade PTC detection and mRNA degradation. If a PTC-bearing allele does escape detection by NMD, protein translation would stop prematurely, and thus no functional full-length PDZD8 protein would be produced.

The lack of PDZD8 protein in human blood limits our ability to detect truncated PDZD8 proteins (31). If produced, truncated PDZD8 would retain the N-terminal TM domain, anchoring the protein to the ER, and the synaptotagmin-like mitochondrial lipid-binding domain involved in dimerization (49). However, it would lack C-terminal regions involved in interaction with another ER TM protein, protrudin (13,34); the phospholipids phosphatidylserine and phosphatidylinositol 4-phosphate, associated with the late endosome/lysosome membrane (13,50); and the late endosomal Rab GTPase, Rab-7a (34,49,51). As an in-frame PTC, p.(Y298∗) is potentially amenable to novel nonsense suppression therapies aimed at suppressing PTCs to restore deficient protein function (52).

To our knowledge, there are no other reports of disease-causing mutations in *PDZD8*. Although *PDZD8* is one of multiple genes occasionally hemizygously lost in distal 10q deletion syndrome, in which ID and dysmorphic features are common (53,54), our finding that syndromic ID is absent from heterozygotes in families A and B suggests that PDZD8 haploinsufficiency is unlikely to have a major impact. A mutation in protrudin (*ZFYVE27*) has previously been identified in spastic paraplegia (55), while mutations in Rab-7a (*RAB7A*) have been identified in Charcot-Marie-Tooth type 2B neuropathy (56, 57, 58, 59).

To assess PDZD8 function in cognition, we undertook a comparative study using targeted interference of *PDZD8* orthologs in two model organisms. *CG10362*-KD fruit flies with KD of the *PDZD8* ortholog showed intact learning and short-term memory in the aversive olfactory conditioning and courtship conditioning assays. However, when tested 48 hours after training in the courtship conditioning assay, *CG10362*-KD males showed deficient long-term courtship-based memory, consistent with interference of PDZD8 impairing long-term memory formation or recall.

*Pdzd8*^*tm1b*^ mice with a PTC (p.F333Nfs1∗) exhibited decreased preweaning viability. Surviving *Pdzd8*^*tm1b*^ mice showed spontaneous repetitive hindlimb jumping, a stereotyped motor behavior with potential relevance to lower-order human motor stereotypies, such as hand flapping, common in ASD (1). High levels of jumping behavior have been reported in *Shank2* null (60), *Sh3rf2* haploinsufficient (61), *Camk2a*-E183V (62), and *Nlgn2* overexpression (63) mice that model genetic risk factors for ASD and in the C58 inbred strain described as a mouse model of autism (64). Approximately 10% of children with ID show or develop autistic symptoms (65), including all affected individuals in families A and B.

Across the 1-hour open field test, *Pdzd8*^*tm1b*^ mice demonstrated reduced habituation—a decrease in response to a stimulus as it becomes familiar—postulated to reflect a deficit in information acquisition (66). In the Barnes maze, *Pdzd8*^*tm1b*^ mice demonstrated reduced memory of the escape hole location in the probe trial, indicative of long-term spatial memory impairment. Our finding that interference of *PDZD8* orthologs resulted in long-term memory deficits in mice and fruit flies provides cross-species substantive evidence linking PDZD8 disruption and cognitive impairment.

In parallel with their hippocampal-dependent spatial memory impairment, *Pdzd8*^*tm1b*^ mice showed diminished LTP evoked by 3× TBS, a maximal induction protocol, in acute hippocampal slices, suggesting that LTP may saturate at lower levels in *Pdzd8*^*tm1b*^ mice. The magnitudes of LTP evoked by the 1× TBS and 1× HFS protocols were, however, not different between genotypes. This suggests that *Pdzd8*^*tm1b*^ mice do not have a global impairment in synaptic function but a subtle hippocampal disruption revealed only when the number of TBS trains is increased or performance on a cognitively demanding task, such as the Barnes maze, is assessed. The reduction in synaptic plasticity at 4 to 6 weeks suggests that plasticity deficits may occur throughout development, and thus learning in the adult may be affected via cumulative synaptic deficits as well as directly.

The ER constitutes a large and important source of Ca^2+^ for various neuronal signaling processes. Ca^2+^ is mobilized from intracellular ER stores upon activation of ryanodine receptors enriched in the dentate gyrus and CA3/4 fields of the hippocampus and/or inositol trisphosphate (IP_3_) receptors enriched in hippocampal CA1 pyramidal cells (67,68). Synaptic plasticity in LTP induction paradigms comparable to our 3× TBS protocol is dependent on the activation of group I metabotropic glutamate receptors (mGluR1 and mGluR5), which results in the stimulation of phospholipase C, leading to IP_3_-mediated Ca^2+^ mobilization from the ER (69, 70, 71, 72). Ca^2+^ mobilized from ER stores transiently reaches concentrations high enough to open the mitochondrial Ca^2+^ uniporter at ER-mitochondria contacts, promoting rapid mitochondrial Ca^2+^ import (73).

In mouse cortical layer II/III pyramidal neurons, synaptic stimulation was shown to induce robust ER Ca^2+^ release and mitochondrial Ca^2+^ uptake in proximal dendrites (15), in which IP_3_ receptors are found (67). This effect was abolished by antagonism of mGluR1, consistent with the known role of mGluR activation in triggering efficient Ca^2+^ release from ER stores (74). KD of *Pdzd8* has been shown to decrease proximity of the ER and mitochondria (14) and decrease mitochondrial Ca^2+^ import evoked by synaptic stimulation, leading to significantly elevated cytosolic Ca^2+^ levels despite unchanged ER Ca^2+^ release (15). The effect of this on Ca^2+^ buffering and adenosine triphosphate synthesis by mitochondria at the synapse is likely to compromise neuronal and synaptic functioning (75). PDZD8 loss of function may have had similar effects following 3× TBS in *Pdzd8*^*tm1b*^ hippocampal slices. Such an impairment of hippocampal neurophysiology that supports spatial memory in *Pdzd8*^*tm1b*^ mice is consistent with the high expression of *Pdzd8* in the mouse hippocampus (15,76). In common with other neurodevelopmental disorders, the cognitive impairment associated with PDZD8 disruption is therefore likely to represent a synaptopathy resulting from synaptic dysfunction (9).

Adult *Pdzd8*^*tm1b*^ mice exhibited decreased absolute brain volume, likely related to their decreased body size, which affected the cerebrum but not the cerebellum and olfactory bulb. Normalizing the volume of each region to absolute brain volume revealed multiple regions with altered relative volumes in *Pdzd8*^*tm1b*^ mice, including the corpus callosum and hippocampus. The relative reduction of the corpus callosum replicates the corpus callosal hypoplasia of the affected female (A.IV.5) in family A. The relative expansion of the hippocampus is comparable to that observed in other mouse models of neurodevelopmental disorders exhibiting cognitive deficits. The heterozygous *Arid1b* knockout mouse model of Coffin-Siris syndrome, a syndromic ID, exhibits body weight and growth rate deficits, reduced total brain volume but hippocampal enlargement, and deficits in novel object recognition (77). The heterozygous *Chd8* mutant mouse model of ASD exhibits an increase in hippocampal volume that is correlated with deficits in contextual fear conditioning (27). Deciphering how PDZD8 disruption affects the tightly orchestrated and intricate processes that determine brain structure will require additional studies.

To summarize, PDZD8 is an ER TM protein required for the extraction of lipids from the ER to late endosomes and lysosomes (13,49) and mitochondrial Ca^2+^ uptake following synaptic transmission–induced Ca^2+^ release from ER stores (15). Our data demonstrate the involvement of homozygous loss-of-function mutations in *PDZD8* in syndromic ID. This knowledge will benefit affected families through genetic counseling and carrier screening and facilitate the genetic diagnosis of other patients. Disrupting the orthologous gene resulted in long-term memory deficits in fruit flies and brain structural alterations, long-term memory deficits, and impaired hippocampal neurophysiology in mice, replicating aspects of the human phenotype and demonstrating a critical role for PDZD8 in brain development and synaptic plasticity. We can use these models to decipher precisely how PDZD8 disruption affects neurodevelopment and synapse function, thus providing insight to the pathophysiological mechanism and potential treatment of this lifelong disability.

## Supplementary Material

Supplementary Material

Key Resources Table

## References

[1] American Psychiatric Association (2013).

[2] World Health Organization (1992).

[3] O’Leary C., Leonard H., Bourke J., D’Antoine H., Bartu A., Bower C. (2013). Intellectual disability: Population-based estimates of the proportion attributable to maternal alcohol use disorder during pregnancy. Dev Med Child Neurol.

[4] Vissers L.E.L.M., Gilissen C., Veltman J.A. (2016). Genetic studies in intellectual disability and related disorders. Nat Rev Genet.

[5] Gilissen C., Hehir-Kwa J.Y., Thung D.T., van de Vorst M., van Bon B.W.M., Willemsen M.H. (2014). Genome sequencing identifies major causes of severe intellectual disability. Nature.

[6] Morton N.E. (1978). Effect of inbreeding on IQ and mental retardation. Proc Natl Acad Sci U S A.

[7] Mir Y.R., Kuchay R.A.H. (2019). Advances in identification of genes involved in autosomal recessive intellectual disability: A brief review. J Med Genet.

[8] Kochinke K., Zweier C., Nijhof B., Fenckova M., Cizek P., Honti F. (2016). Systematic phenomics analysis deconvolutes genes mutated in intellectual disability into biologically coherent modules. Am J Hum Genet.

[9] Volk L., Chiu S.L., Sharma K., Huganir R.L. (2015). Glutamate synapses in human cognitive disorders. Annu Rev Neurosci.

[10] Torres V.I., Vallejo D., Inestrosa N.C. (2017). Emerging synaptic molecules as candidates in the etiology of neurological disorders. Neural Plast.

[11] Moretto E., Murru L., Martano G., Sassone J., Passafaro M. (2018). Glutamatergic synapses in neurodevelopmental disorders. Prog Neuropsychopharmacol Biol Psychiatry.

[12] Willemsen M.H., Kleefstra T. (2014). Making headway with genetic diagnostics of intellectual disabilities. Clin Genet.

[13] Shirane M., Wada M., Morita K., Hayashi N., Kunimatsu R., Matsumoto Y. (2020). Protrudin and PDZD8 contribute to neuronal integrity by promoting lipid extraction required for endosome maturation. Nat Commun.

[14] Hertlein V., Flores-Romero H., Das K.K., Fischer S., Heunemann M., Calleja-Felipe M. (2020). MERLIN: A novel BRET-based proximity biosensor for studying mitochondria-ER contact sites. Life Sci Alliance.

[15] Hirabayashi Y., Kwon S.K., Paek H., Pernice W.M., Paul M.A., Lee J. (2017). ER-mitochondria tethering by PDZD8 regulates Ca^2+^ dynamics in mammalian neurons. Science.

[16] Al-Amri A., Al Saegh A., Al-Mamari W., El-Asrag M.E., Ivorra J.L., Cardno A.G. (2016). Homozygous single base deletion in TUSC3 causes intellectual disability with developmental delay in an Omani family. Am J Med Genet A.

[17] Dietzl G., Chen D., Schnorrer F., Su K.C., Barinova Y., Fellner M. (2007). A genome-wide transgenic RNAi library for conditional gene inactivation in Drosophila. Nature.

[18] McBride S.M., Giuliani G., Choi C., Krause P., Correale D., Watson K. (1999). Mushroom body ablation impairs short-term memory and long-term memory of courtship conditioning in Drosophila melanogaster. Neuron.

[19] Mery F., Kawecki T.J. (2005). A cost of long-term memory in Drosophila. Science.

[20] Koemans T.S., Oppitz C., Donders R.A.T., van Bokhoven H., Schenck A., Keleman K., Kramer J.M. (2017). Drosophila courtship conditioning as a measure of learning and memory. J Vis Exp.

[21] Friedel R.H., Seisenberger C., Kaloff C., Wurst W. (2007). EUCOMM—The European conditional mouse mutagenesis program. Brief Funct Genomic Proteomic.

[22] Dachtler J., Glasper J., Cohen R.N., Ivorra J.L., Swiffen D.J., Jackson A.J. (2014). Deletion of α-neurexin II results in autism-related behaviors in mice. Transl Psychiatry.

[23] Kirshenbaum G.S., Dachtler J., Roder J.C., Clapcote S.J. (2015). Characterization of cognitive deficits in mice with an alternating hemiplegia-linked mutation. Behav Neurosci.

[24] Illouz T., Madar R., Clague C., Griffioen K.J., Louzoun Y., Okun E. (2016). Unbiased classification of spatial strategies in the Barnes maze. Bioinformatics.

[25] Riedel G., Robinson L., Crouch B. (2018). Spatial learning and flexibility in 129S2/SvHsd and C57BL/6J mouse strains using different variants of the Barnes maze. Behav Pharmacol.

[26] Dennis S.H., Pasqui F., Colvin E.M., Sanger H., Mogg A.J., Felder C.C. (2016). Activation of muscarinic M1 acetylcholine receptors induces long-term potentiation in the hippocampus. Cereb Cortex.

[27] Gompers A.L., Su-Feher L., Ellegood J., Copping N.A., Riyadh M.A., Stradleigh T.W. (2017). Germline Chd8 haploinsufficiency alters brain development in mouse. Nat Neurosci.

[28] Karczewski K.J., Francioli L.C., Tiao G., Cummings B.B., Alföldi J., Wang Q. (2020). The mutational constraint spectrum quantified from variation in 141,456 humans [published correction appears in Nature 2021; 590:E53] [published correction appears in Nature 2021; 597:E3–E4]. Nature.

[29] Adzhubei I., Jordan D.M., Sunyaev S.R. (2013). Predicting functional effect of human missense mutations using PolyPhen-2. Curr Protoc Hum Genet.

[30] Ng P.C., Henikoff S. (2003). SIFT: Predicting amino acid changes that affect protein function. Nucleic Acids Res.

[31] Uhlén M., Fagerberg L., Hallström B.M., Lindskog C., Oksvold P., Mardinoglu A. (2015). Proteomics. Tissue-based map of the human proteome. Science.

[32] Smedley D., Smith K.R., Martin A., Thomas E.A., McDonagh E.M., 100,000 Genomes Project Pilot Investigators (2021). 100,000 Genomes pilot on rare-disease diagnosis in health care—Preliminary report. N Engl J Med.

[33] Nakada C., Tsukamoto Y., Oka A., Nonaka I., Sato K., Mori S. (2004). Altered expression of ARPP protein in skeletal muscles of patients with muscular dystrophy, congenital myopathy and spinal muscular atrophy. Pathobiology.

[34] Elbaz-Alon Y., Guo Y., Segev N., Harel M., Quinnell D.E., Geiger T. (2020). PDZD8 interacts with Protrudin and Rab7 at ER-late endosome membrane contact sites associated with mitochondria. Nat Commun.

[35] UniProt Consortium (2021). UniProt: The universal protein Knowledgebase in 2021. Nucleic Acids Res.

[36] Supek F., Lehner B., Lindeboom R.G.H. (2021). To NMD or not to NMD: Nonsense-mediated mRNA decay in cancer and other genetic diseases. Trends Genet.

[37] Bakken T.E., Jorstad N.L., Hu Q., Lake B.B., Tian W., Kalmbach B.E. (2021). Comparative cellular analysis of motor cortex in human, marmoset and mouse. Nature.

[38] Miller J.A., Ding S.L., Sunkin S.M., Smith K.A., Ng L., Szafer A. (2014). Transcriptional landscape of the prenatal human brain. Nature.

[39] Lee I., Hong W. (2006). Diverse membrane-associated proteins contain a novel SMP domain. FASEB J.

[40] Leader D.P., Krause S.A., Pandit A., Davies S.A., Dow J.A.T. (2018). FlyAtlas 2: A new version of the Drosophila melanogaster expression atlas with RNA-Seq, miRNA-Seq and sex-specific data. Nucleic Acids Res.

[41] Bolivar V.J., Caldarone B.J., Reilly A.A., Flaherty L. (2000). Habituation of activity in an open field: A survey of inbred strains and F_1_ hybrids. Behav Genet.

[42] Gallitano-Mendel A., Izumi Y., Tokuda K., Zorumski C.F., Howell M.P., Muglia L.J. (2007). The immediate early gene early growth response gene 3 mediates adaptation to stress and novelty. Neuroscience.

[43] Rodgers R.J., Johnson N.J. (1995). Factor analysis of spatiotemporal and ethological measures in the murine elevated plus-maze test of anxiety. Pharmacol Biochem Behav.

[44] Griebel G., Rodgers R.J., Perrault G., Sanger D.J. (1997). Risk assessment behaviour: Evaluation of utility in the study of 5-HT-related drugs in the rat elevated plus-maze test. Pharmacol Biochem Behav.

[45] Weiss S.M., Wadsworth G., Fletcher A., Dourish C.T. (1998). Utility of ethological analysis to overcome locomotor confounds in elevated maze models of anxiety. Neurosci Biobehav Rev.

[46] Bliss T.V., Collingridge G.L. (1993). A synaptic model of memory: Long-term potentiation in the hippocampus. Nature.

[47] Zucker R.S., Regehr W.G. (2002). Short-term synaptic plasticity. Annu Rev Physiol.

[48] Cirulli E.T., Heinzen E.L., Dietrich F.S., Shianna K.V., Singh A., Maia J.M. (2011). A whole-genome analysis of premature termination codons. Genomics.

[49] Gao Y., Xiong J., Chu Q.Z., Ji W.K. (2022). PDZD8-mediated lipid transfer at contacts between the ER and late endosomes/lysosomes is required for neurite outgrowth. J Cell Sci.

[50] Hammond G.R.V., Machner M.P., Balla T. (2014). A novel probe for phosphatidylinositol 4-phosphate reveals multiple pools beyond the Golgi. J Cell Biol.

[51] Guillén-Samander A., Bian X., De Camilli P. (2019). PDZD8 mediates a Rab7-dependent interaction of the ER with late endosomes and lysosomes. Proc Natl Acad Sci U S A.

[52] Martins-Dias P., Romão L. (2021). Nonsense suppression therapies in human genetic diseases. Cell Mol Life Sci.

[53] Yatsenko S.A., Kruer M.C., Bader P.I., Corzo D., Schuette J., Keegan C.E. (2009). Identification of critical regions for clinical features of distal 10q deletion syndrome. Clin Genet.

[54] Nandyal R., Hagan S., Sandhu T. (2019). Neonate with 10q interstitial deletion within the long arm of chromosome 10–A case report and literature review. Int J Case Rep.

[55] Mannan A.U., Krawen P., Sauter S.M., Boehm J., Chronowska A., Paulus W. (2006). ZFYVE27 (SPG33), a novel spastin-binding protein, is mutated in hereditary spastic paraplegia. Am J Hum Genet.

[56] Verhoeven K., De Jonghe P., Coen K., Verpoorten N., Auer-Grumbach M., Kwon J.M. (2003). Mutations in the small GTP-ase late endosomal protein RAB7 cause Charcot-Marie-Tooth type 2B neuropathy. Am J Hum Genet.

[57] Houlden H., King R.H.M., Muddle J.R., Warner T.T., Reilly M.M., Orrell R.W., Ginsberg L. (2004). A novel RAB7 mutation associated with ulcero-mutilating neuropathy. Ann Neurol.

[58] Meggouh F., Bienfait H.M.E., Weterman M.A.J., de Visser M., Baas F. (2006). Charcot-Marie-Tooth disease due to a de novo mutation of the RAB7 gene. Neurology.

[59] Rotthier A., Baets J., De Vriendt E., Jacobs A., Auer-Grumbach M., Lévy N. (2009). Genes for hereditary sensory and autonomic neuropathies: A genotype-phenotype correlation. Brain.

[60] Won H., Lee H.R., Gee H.Y., Mah W., Kim J.I., Lee J. (2012). Autistic-like social behaviour in Shank2-mutant mice improved by restoring NMDA receptor function. Nature.

[61] Wang S., Tan N., Zhu X., Yao M., Wang Y., Zhang X., Xu Z. (2018). Sh3rf2 haploinsufficiency leads to unilateral neuronal development deficits and autistic-like behaviors in mice. Cell Rep.

[62] Stephenson J.R., Wang X., Perfitt T.L., Parrish W.P., Shonesy B.C., Marks C.R. (2017). A novel human CAMK2A mutation disrupts dendritic morphology and synaptic transmission, and causes ASD-related behaviors. J Neurosci.

[63] Hines R.M., Wu L., Hines D.J., Steenland H., Mansour S., Dahlhaus R. (2008). Synaptic imbalance, stereotypies, and impaired social interactions in mice with altered neuroligin 2 expression. J Neurosci.

[64] Silverman J.L., Smith D.G., Sukoff Rizzo S.J., Karras M.N., Turner S.M., Tolu S.S. (2012). Negative allosteric modulation of the mGluR5 receptor reduces repetitive behaviors and rescues social deficits in mouse models of autism. Sci Transl Med.

[65] Oeseburg B., Dijkstra G.J., Groothoff J.W., Reijneveld S.A., Jansen D.E.M.C. (2011). Prevalence of chronic health conditions in children with intellectual disability: A systematic literature review. Intellect Dev Disabil.

[66] Platel A., Porsolt R.D. (1982). Habituation of exploratory activity in mice: A screening test for memory enhancing drugs. Psychopharmacology (Berl).

[67] Sharp A.H., McPherson P.S., Dawson T.M., Aoki C., Campbell K.P., Snyder S.H. (1993). Differential immunohistochemical localization of inositol 1,4,5-trisphosphate- and ryanodine-sensitive Ca^2+^ release channels in rat brain. J Neurosci.

[68] Maggio N., Vlachos A. (2014). Synaptic plasticity at the interface of health and disease: New insights on the role of endoplasmic reticulum intracellular calcium stores. Neuroscience.

[69] Raymond C.R., Thompson V.L., Tate W.P., Abraham W.C. (2000). Metabotropic glutamate receptors trigger homosynaptic protein synthesis to prolong long-term potentiation. J Neurosci.

[70] Raymond C.R., Redman S.J. (2002). Different calcium sources are narrowly tuned to the induction of different forms of LTP. J Neurophysiol.

[71] Raymond C.R., Redman S.J. (2006). Spatial segregation of neuronal calcium signals encodes different forms of LTP in rat hippocampus. J Physiol.

[72] Shalin S.C., Hernandez C.M., Dougherty M.K., Morrison D.K., Sweatt J.D. (2006). Kinase suppressor of Ras1 compartmentalizes hippocampal signal transduction and subserves synaptic plasticity and memory formation. Neuron.

[73] Patron M., Checchetto V., Raffaello A., Teardo E., Vecellio Reane D., Mantoan M. (2014). MICU1 and MICU2 finely tune the mitochondrial Ca^2+^ uniporter by exerting opposite effects on MCU activity. Mol Cell.

[74] Frenguelli B.G., Potier B., Slater N.T., Alford S., Collingridge G.L. (1993). Metabotropic glutamate receptors and calcium signalling in dendrites of hippocampal CA1 neurones. Neuropharmacology.

[75] Datta S., Jaiswal M. (2021). Mitochondrial calcium at the synapse. Mitochondrion.

[76] Lein E.S., Hawrylycz M.J., Ao N., Ayres M., Bensinger A., Bernard A. (2007). Genome-wide atlas of gene expression in the adult mouse brain. Nature.

[77] Ellegood J., Petkova S.P., Kinman A., Qiu L.R., Adhikari A., Wade A.A. (2021). Neuroanatomy and behavior in mice with a haploinsufficiency of AT-rich interactive domain 1B (ARID1B) throughout development. Mol Autism.

