# Supplementary material for: PDZD8 Disruption Causes Cognitive Impairment in Humans, Mice, and Fruit Flies
Source: Biol Psychiatry. 2022 Aug 15;92(4):323–34. doi: 10.1016/j.biopsych.2021.12.017 (PMC9302898; doi:10.1016/j.biopsych.2021.12.017)
Supplement: Supplementary Material [file mmc1.pdf]

PDZD8 Disruption Causes Cognitive Impairment in Humans, Mice, and Fruit Flies

SUPPLEMENTAL INFORMATION

SUPPLEMENTAL TABLES

Table S1. Mutation identified in *ANKRD2*.

Family	Ethnicity	Genotype	ANKRD2 modification	Nucleotide change	gnomAD ID	Frequency in gnomAD[1]	CADD score	PolyPhen	SIFT
A	Omani	Homozygous	p.(R328W)	c.982C>T	10-99343381-C-T	0.00001961	34	possibly damaging	deleterious

Nucleotide and residue numbering are based on NM_020349.
[1]gnomAD v2.1.1 (controls): 60,146 samples from individuals who were not selected as a case in a case/control study of common disease.

SUPPLEMENTAL FIGURES

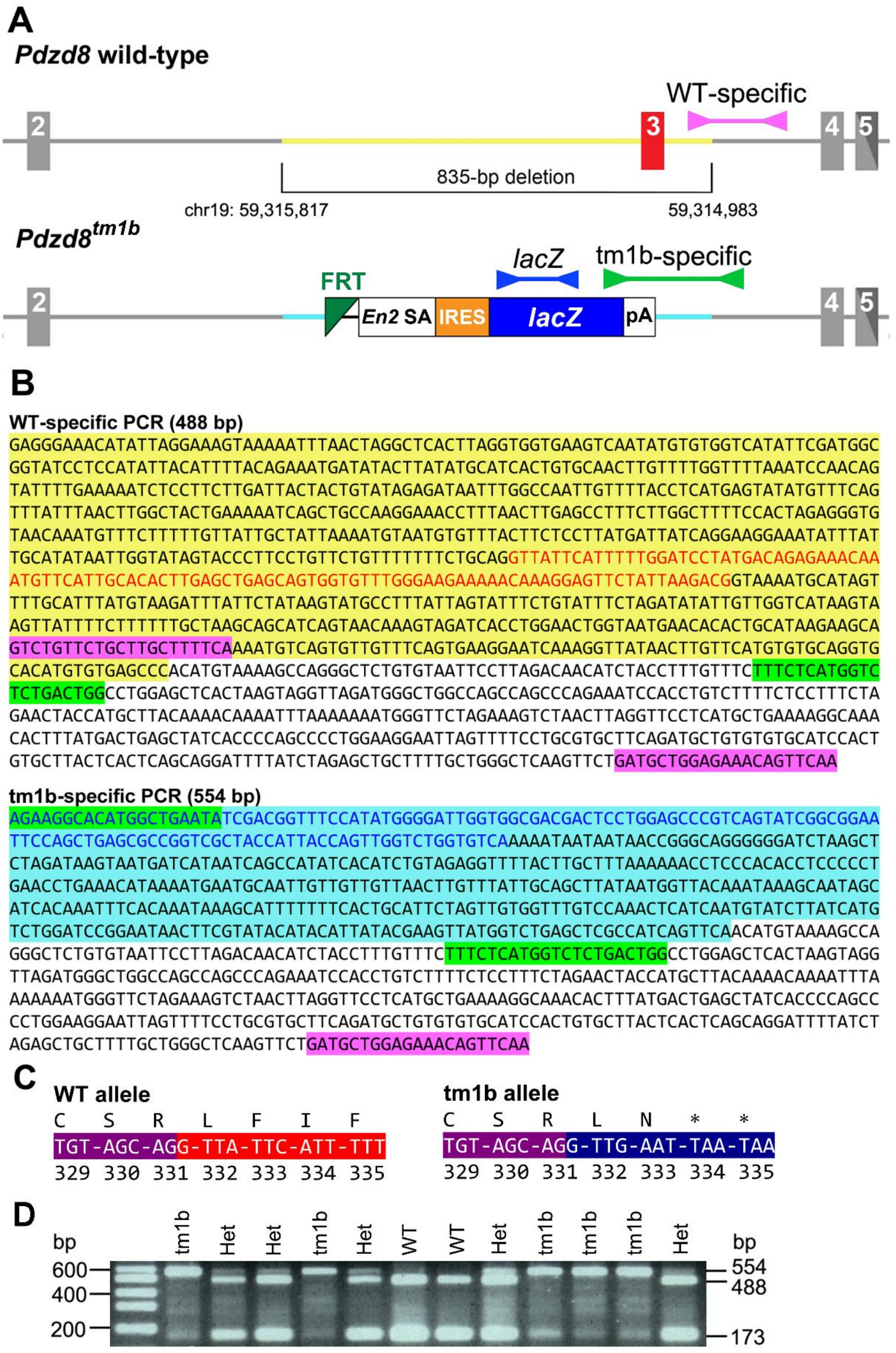

Figure S1. Genotyping of *Pdzd8* reporter-tagged deletion allele. **(A)** Top, *Pdzd8* WT allele showing 835-bp sequence (chr19:59,315,817-59,314,983; GRCm38) (yellow) including exon 3 (red box) that is deleted from *Pdzd8*^{tm1b} allele. Bottom, *Pdzd8*^{tm1b} allele in which targeted 835 bp including exon 3 has been replaced by a *lacZ* expression cassette. Bow

ties indicate location of WT-specific (magenta), *Pdzd8*^{tm1b}-specific (green) and *lacZ* (blue) PCR assays. **(B)** Top, WT-specific PCR assay showing location of WT-specific forward (5'-GTC TGT TCT GCT TGC TTT TCA-3') and reverse (5'-TTG AAC TGT TTC TCC AGC ATC-3') primers (magenta background; 488-bp amplicon), *Pdzd8*^{tm1b}-specific forward primer (green background), and 835 bp including exon 3 (red text) deleted from *Pdzd8*^{tm1b} allele (yellow background). Bottom, *Pdzd8*^{tm1b}-specific PCR assay showing location of *Pdzd8*^{tm1b}-specific forward (5'-AGA AGG CAC ATG GCT GAA TA-3') and reverse (5'-CCA GTC AGA GAC CAT GAG AAA-3') primers (green background; 544-bp amplicon), WT-specific reverse primer (magenta background), 3' end of *lacZ* cDNA (blue text), and 3' end of *lacZ* expression cassette (light blue background). **(C)** Frameshift that changes F333 and I334 in WT allele (left) to N333 and *334 in *Pdzd8*^{tm1b} allele (p.F333Nfs1*) (right). Exon 2 (purple background); exon 3 (red background); exon 4 (dark blue background). **(D)** *Pdzd8* multiplex PCR genotyping gel. The sizes of the DNA ladder fragments (left) and PCR products (right) are indicated. The 173-bp fragment is the product of the WT-specific forward primer and *Pdzd8*^{tm1b}-specific reverse primer. FRT, flippase recognition target; *En2* SA, mouse *En2* (engrailed-2) intron including splice acceptor; IRES, internal ribosome entry site of encephalomyocarditis virus (ECMV); *lacZ*, *E. coli lacZ* gene encoding β-galactosidase; pA, Simian virus 40 (SV40) polyadenylation signal; F, phenylalanine; I, isoleucine; N, asparagine; *, stop; bp, base pairs; tm1b, homozygous; Het, heterozygous; WT, wild type.

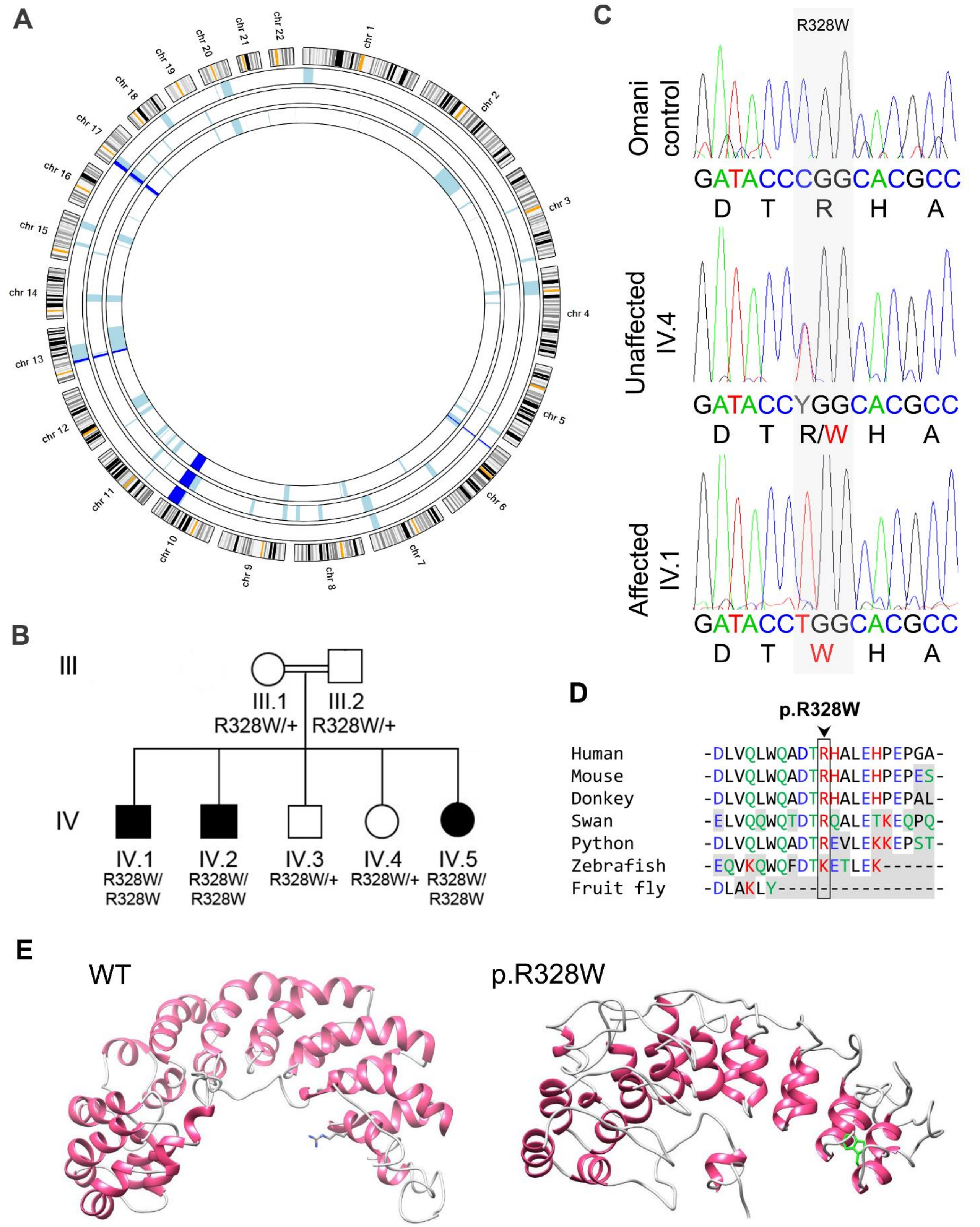

Figure S2. Homozygosity mapping and identification of *ANKRD2* variant in family A. **(A)** AgileMultiIdeogram representation of the homozygosity mapping data from family A using the GRCh38 reference assembly. **(B)** Pedigree of nuclear family A showing co-segregation of *ANKRD2* p.(R328W) homozygosity with neurodevelopmental phenotype in three affected siblings (represented by filled symbols). **(C)** Sanger sequence chromatograms showing the

ANKRD2 c.982C>T (p.R328W) variant identified in family A. **(D)** Partial protein sequence alignment of human ANKRD2 and orthologs showing conservation of the residues affected by the p.(R328W) variant in family A. Residues disparate between human and the other species have a gray background. Accession numbers for ANKRD2 protein are human (*Homo sapiens*) NP_065082; mouse (*Mus musculus*) NP_064417; donkey (*Equus asinus*) XP_014718200; swan (*Cygnus atratus*) XP_035400949; python (*Python bivittatus*) XP_007419962; zebrafish (*Danio rerio*) XP_021336623; fruit fly (*Drosophila melanogaster*): NP_001097672. Amino acid color code: black (nonpolar), green (uncharged polar), red (basic), blue (acidic). **(E)** Structural modeling of ANKRD2 protein. WT ANKRD2 (left). R328 is shown at the underside of the protein with the sidechain projecting outwards (represented as sticks). R328 is located towards the centre of the last of multiple repeats of short alpha helices (7 residues in length) that orientate in a single plane, facilitating stacking. p.R328W ANKRD2 (right). W328 is shown at the underside of the protein, with the sidechain (represented as sticks) in a more buried location compared to R328. The p.R328W variant changes the local orientation of the alpha helices and introduces slightly longer stretches of beta turn between the helices. It is likely that these differences, and the loss of the arginine residue itself along with the associated electrostatic interactions, has an impact on oligomerization or on interactions with other titin filament proteins.

```
MGLLLMILASAVLGSFLTLLAQFFLLYRRQPEPPADEAARAGEGFRYIKPVPGLLLREYLYGGGRDEEPSGAAPEGGATP
TAAPETPAPPTRETCYFLNATILFLFRELRDTALTRRWVTKKIKVEFEELLQTKTAGRLLEGLSLRDVFLGETVPFIKTI
RLVRPVVPSATGEPDGPEGEALPAACPEELAFEAEVEYNGGFHLAIDVDLVFGKSAYLFVKLSRVVGRLRLVFTRVPFTH
WFFSFVEDPLIDFEVRSQFEGRPMPQLTSIIVNQLKKIIKRKHTLPNYKIRFKPFFPYQTLQGFEEDEEHIHIQQWALTE
GRLKVTLLECSRLLIFGSYDREANVHCTLELSSSVWEEKQRSSIKTVELIKGNLQSVGLTLRLVQSTDGYAGHVIIETVA
PNSPAAIADLQRGDRLIAIGGVKITSTLQVLKLIKQAGDRVLVYYERPVGQSNQGAVLQDNFGQLEENFLSSSCQSGYEE
EAAGLTVDTESRELDSEFEDLASDVRAQNEFKDEAQSLSHSPKRVPTTLSIKPLGAISPVLNRKLAVGSHPLPPKIQSKD
GNKPPPLKTSEITDPAQVSKPTQGSAFKPPVPPRPQAKVPLPSADAPNQAEPDVLVEKPEKVVPPPLVDKSAEKQAKNVD
AIDDAAAPKQFLAKQEVAKDVTSETSCPTKDSSDDRQTWESSEILYRNKLGKWTRTRASCLFDIEACHRYLNIALWCRDP
FKLGGLICLGHVSLKLEDVALGCLATSNTEYLSKLRLEAPSPKAIVTRTALRNLSMQKGFNDKFCYGDITIHFKYLKEGE
SDHHVVTNVEKEKEPHLVEEVSVLPKEEQFVGQMGLTENKHSFQDTQFQNPTWCDYCKKKVWTKAASQCMFCAYVCHKKC
QEKCLAETSVCGATDRRIDRTLKNLRLEGQETLLGLPPRVDAEASKSVNKTTGLTRHIINTSSRLLNLRQVSKTRLSEPG
TDLVEPSPKHTPNTSDNEGSDTEVCGPNSPSKRGNSTGIKLVRKEGGLDDSVFIAVKEIGRDLYRGLPTEERIQKLEFML
DKLQNEIDQELEHNNSLVREEKETTDTRKKSLLSAALAKSGERLQALTLLMIHYRAGIEDIETLESLSLDQHSKKISKYT
DDTEEDLDNEISQLIDSQPFSSISDDLFGPSESV
```

TM SMP PDZ PR C1 CC

Figure S3. Location of the p.(Y298*) and p.(S733*) variants within protein sequence and domain organization of human PDZD8 (Q8NEN9). The residues affected by p.(Y298*) and p.(S733*) are shown in red text. The residues (L334 & I335) corresponding to F333 and I334 affected by p.F333Nfs1* in mouse PDZD8 are shown in blue text. The 50-aa epitope (935–984 aa) of antibody PA5-46771 to PDZD8 is underlined. TM, transmembrane; SMP, synaptotagmin-like mitochondrial lipid-binding; PDZ, PSD-95/DlgA/ZO-1-like; PR, proline-rich; C1, phorbol-ester/diacylglycerol-binding; CC, coiled-coil.

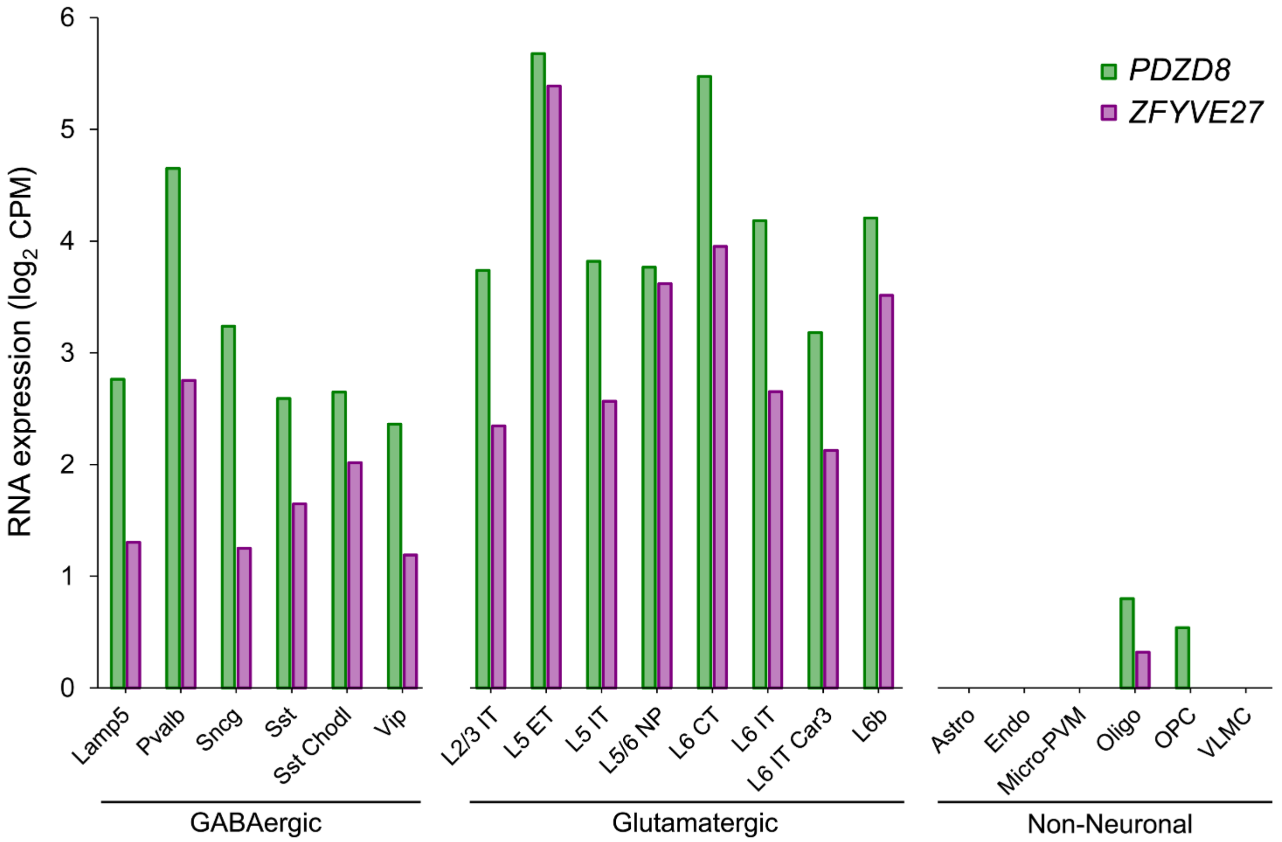

Figure S4. *PDZD8* mRNA expression in different cell types in adult human primary motor cortex. RNA-sequencing data from the Allen Cell Types Database for *PDZD8* and *ZFYVE27* (protrudin) among 20 types of GABAergic and glutamatergic neurons and non-neuronal cells, presented as trimmed mean $\log_2$ CPM (counts per million reads mapped), averaged after excluding the 25% highest and 25% lowest expression values (1). Lamp5, lysosomal-associated membrane protein 5; Pvalb, parvalbumin; Sncg, γ-synuclein; Sst, somatostatin; Chodl, chondrolectin; Vip, vasoactive intestinal peptide; L, cortical layer; IT, intratelencephalic; ET, extratelencephalic; NP, near-projecting; CT, corticothalamic; Car3, carbonic anhydrase 3; Astro, astrocyte; Endo, endothelial cell; Microglia-PVM, microglia / perivascular macrophage; Oligo, oligodendrocyte; OPC, oligodendrocyte precursor cell; VLMC, vascular leptomeningeal cell.

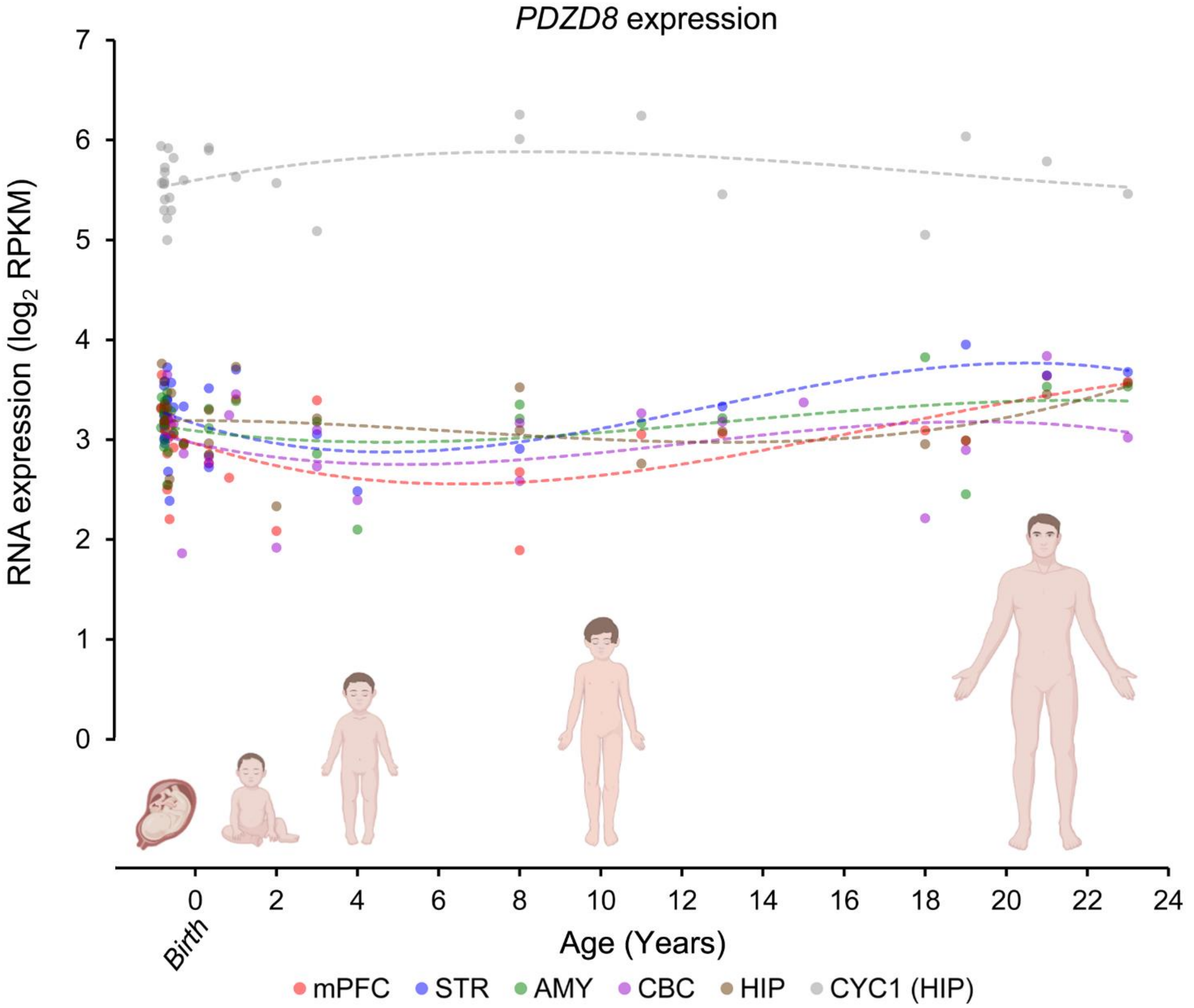

Figure S5. *PDZD8* mRNA expression in the developing human brain. Raw RNA-sequencing data from BrainSpan project (2) for *PDZD8* and *CYC1* reference gene (3), presented as log_2 RPKM (reads per kilobase exon per million mapped reads) and a polynomial fitted to average across ages (broken line), from 8 weeks post conception (wpc) to early adulthood (23 years). Brain regions include medial prefrontal cortex (mPFC), striatum (STR), amygdaloid complex (AMY), cerebellar cortex (CBC), and hippocampus (HIP).

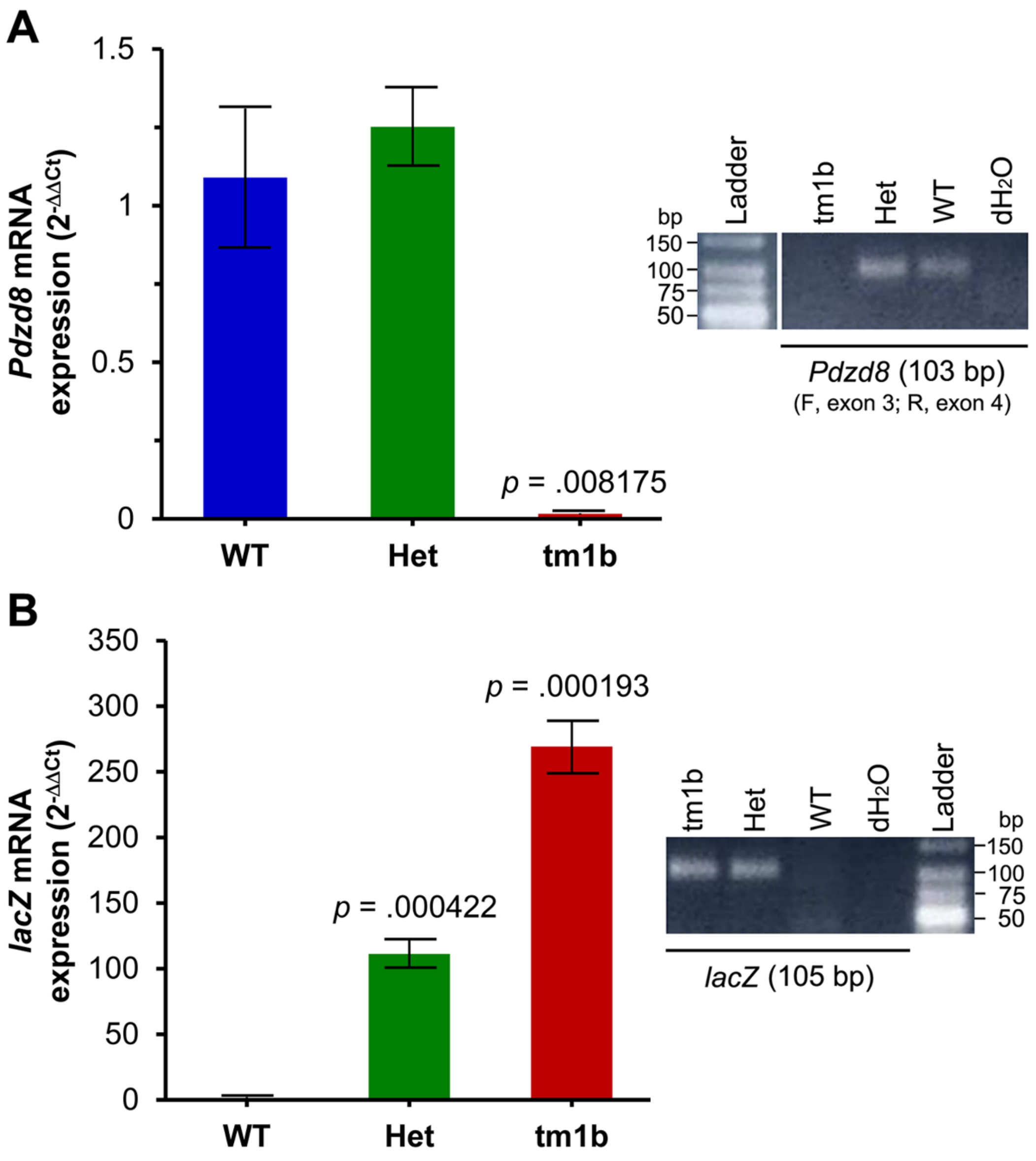

Figure S6. *Pdzd8* mRNA expression in *Pdzd8*^{tm1b} mouse brain. **(A)** Absence of 103-bp band of *Pdzd8* mRNA including exon 3 in *Pdzd8*^{tm1b} whole brain. **(B)** Absence of 105-bp band of *lacZ* mRNA in WT whole brain. Gene mRNA expression is presented as fold change ± SEM, calculated via the $2^{-\Delta\Delta Ct}$ method (4), relative to 120-bp band of *Hprt* mRNA (reference gene). Student's *t*-test detected significant differences in *Pdzd8*^{tm1b} (tm1b) and heterozygous (Het) versus WT male mice (n = 5/genotype). Normalizing to the *B2m* reference gene gave similar results.

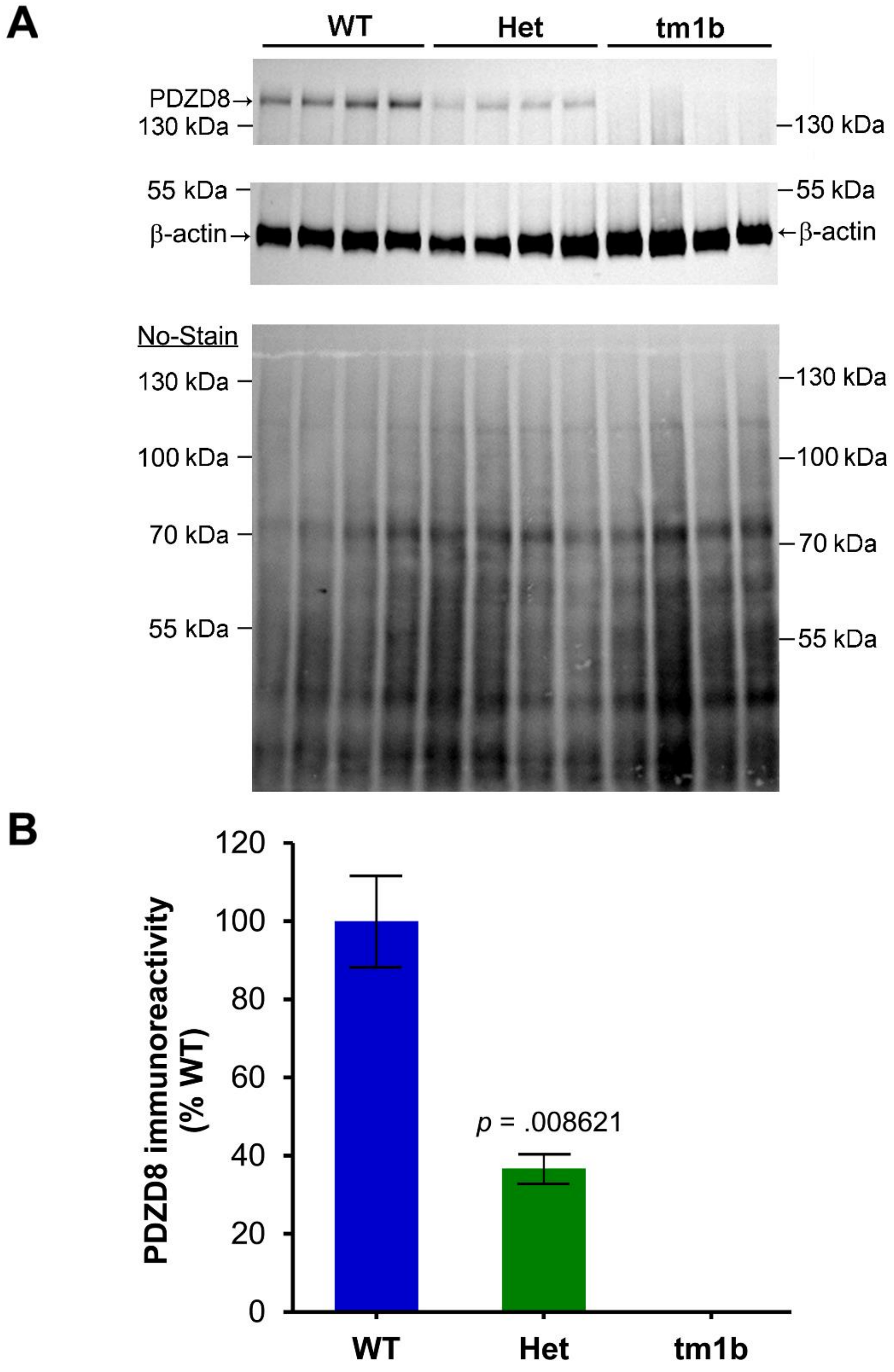

Figure S7. PDZD8 abundance in *Pdzd8^{tm1b}* mouse brain. **(A)** Western blots of protein from whole brain probed with antibody PA5-46771 to PDZD8, showing absence of the ~140-kDa protein from *Pdzd8^{tm1b}* (tm1b) samples (*Top*), and antibody A1978 to β-actin (loading control) (*Middle*). Visualization of all proteins on the post-transfer membrane by No-Stain Protein Labeling Reagent (Invitrogen, Paisley, UK) (*Bottom*). Samples were loaded alongside PageRuler Plus Prestained Protein Ladder (Thermo Scientific, Loughborough, UK). **(B)** PDZD8 immunoreactivity, normalized to β-actin, showing 63.24 ± 3.84% less abundance of PDZD8 in heterozygous (Het) relative to WT samples (n = 4/genotype) (t-test: $p < .01$ versus WT).

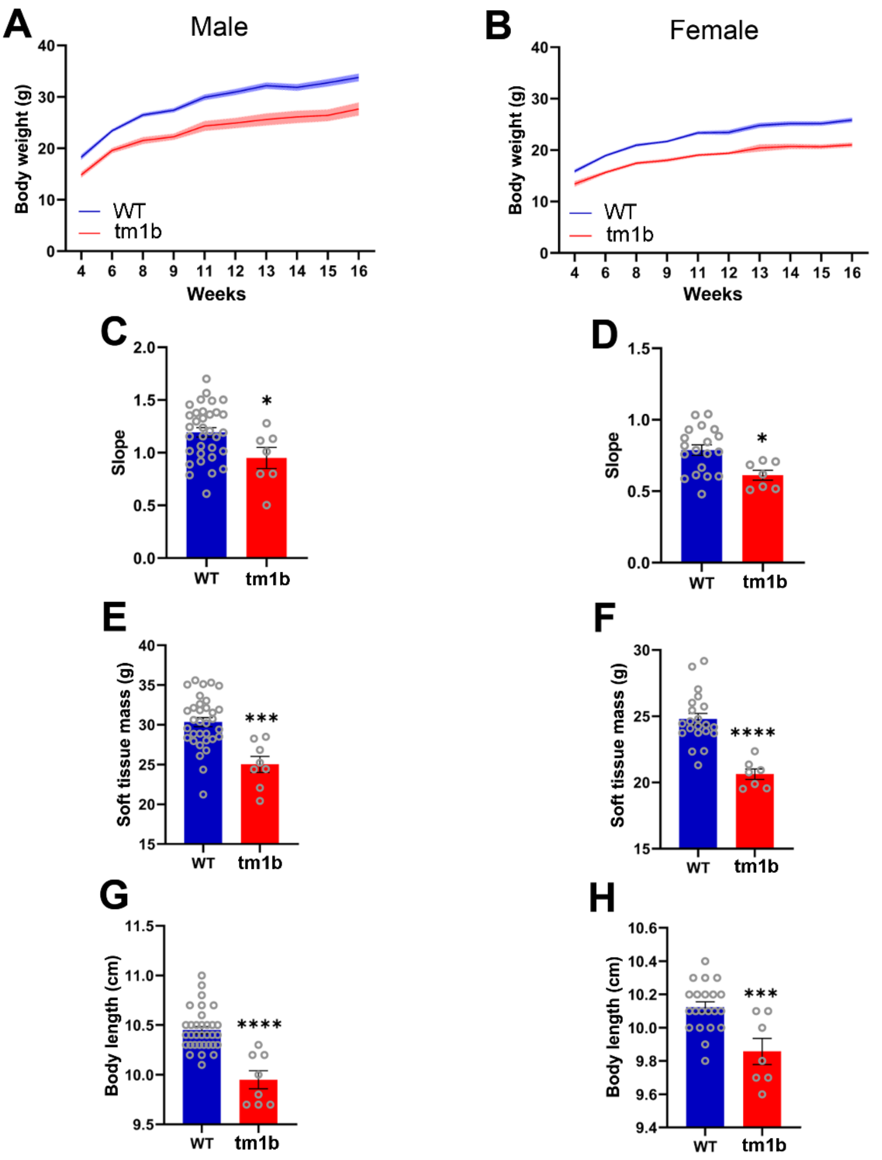

Figure S8. Growth restriction of *Pdzd8^tm1b^* mice. **(A)** Body weight (g) of male *Pdzd8^tm1b^* (n = 7) and WT (n = 33) mice at 4–16 weeks of age (two-way repeated measures ANOVA, genotype: $F_{1,38}$ = 19.90, p < .001; week: $F_{1.60,61.12}$ = 294.85, p < .001; interaction: $F_{1.60,61.12}$ = 4.17, p = .027). At 16 weeks, male *Pdzd8^tm1b^* mice showed an 18.14 ± 3.79% reduction in body weight. **(B)** Body weight (g) of female *Pdzd8^tm1b^* (n = 7) and WT (n = 19) mice at 4–16 weeks of age (two-way repeated measures ANOVA, genotype: $F_{1,24}$ = 49.52, p < .001; week: $F_{3.21,77.12}$ = 220.919, p < .001; interaction: $F_{3.21,77.12}$ = 3.499, p = .017). At 16 weeks, female *Pdzd8^tm1b^* mice showed an 18.67 ± 1.64% reduction in body weight. **(C)** Slope of growth curve of male *Pdzd8^tm1b^* (0.9486 ± 0.09978) and WT (1.193 ± 0.04484) mice (independent t-test: t_{38} = 2.26, p = .029). **(D)** Slope of growth curve of female *Pdzd8^tm1b^* (0.6118 ± 0.03495) and WT (0.787 ± 0.03686) mice (independent t-test: t_{24} = 2.70, p = .01). **(E)** Soft tissue mass (g) of male *Pdzd8^tm1b^* (n = 8; 25.02 ± 1.004) and WT (n = 33; 30.34 ± 0.5714) mice at 14 weeks of age (independent t-test: t_{39} = 4.20, p < .001). **(F)** Soft tissue mass (g) of female

Pdzd8^{tm1b} (n = 7; 20.63 ± 0.3954) and WT (n = 21; 24.78 ± 0.4238) mice at 14 weeks of age (independent t-test: t_{26} = 5.363, p < .001). **(G)** Body length (cm) of male *Pdzd8^{tm1b}* (9.95 ± 0.09063) and WT (10.45 ± 0.03592) mice at 14 weeks of age (Mann-Whitney U test: U = 13, p < .001). **(H)** Body length (cm) of female *Pdzd8^{tm1b}* (9.857 ± 0.07825) and WT (10.12 ± 0.03155) mice at 14 weeks of age (independent t-test: t_{26} = 3.791, p < .001). Data are plotted as mean ± SEM. *p < .05; ***p < .001; ****p < .0001 vs. WT.

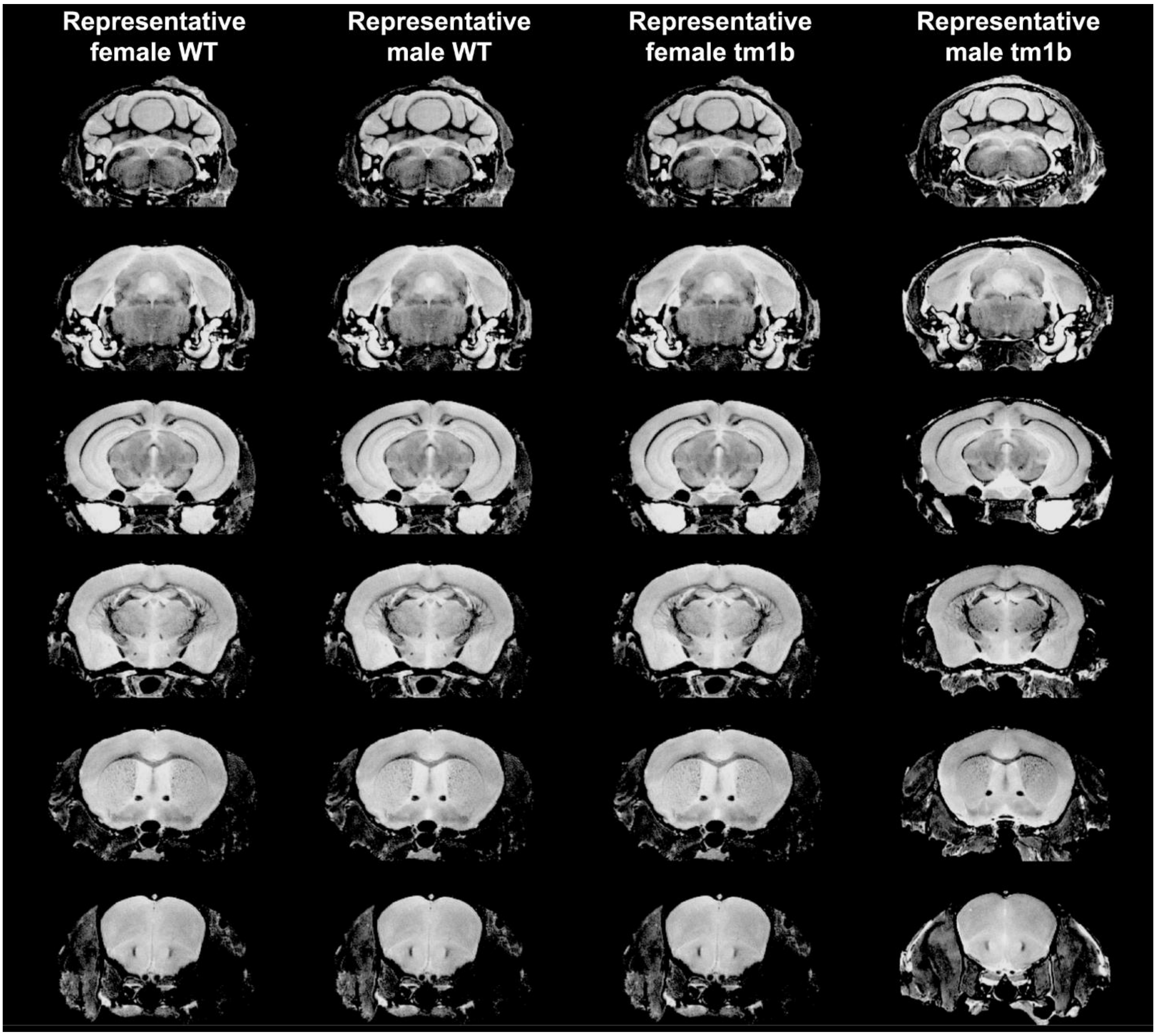

Figure S9. Representative structural images of brains from *Pdzd8*^{tm1b} and WT mice of each sex. Raw structural images realigned with a LSQ6 (least squares with 6 degrees-of-freedom) transformation so they are in the same space.

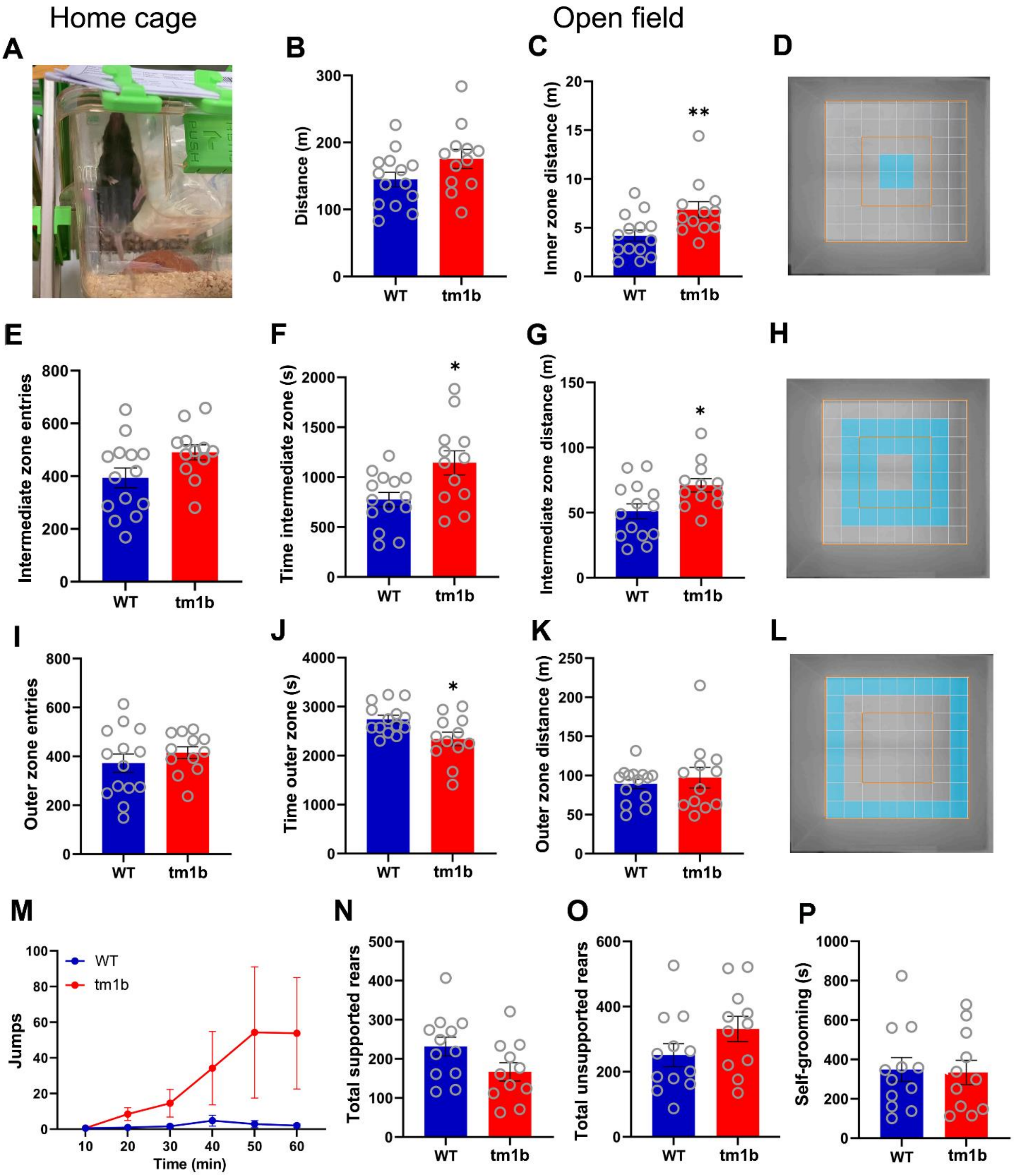

Figure S10. Behavioral differences of $Pdzd8^{tm1b}$ mice in home cage and open field (OF). **(A)** Representative image of hindlimb jumping by $Pdzd8^{tm1b}$ mouse in home cage. **(B)** Total distance travelled (m) by $Pdzd8^{tm1b}$ (n = 12, 175.5 ± 14.26) and WT (n = 14, 144.8 ± 10.81) mice in OF (t_{24} = 1.743, p = .094). **(C)** Distance travelled (m) by $Pdzd8^{tm1b}$ (6.853 ± 0.8227) and WT (4.183 ± 0.5713) mice in OF inner zone (t_{24} = -2.933, p = .007). **(D)** OF inner zone (100 cm^2). **(E)** No. entries by $Pdzd8^{tm1b}$ (490.3 ± 28.9) and WT (393.6 ± 37.56) mice to OF intermediate zone (t_{24} = 1.987, p = .058). **(F)** Time (s) spent by $Pdzd8^{tm1b}$ (1144.0 ± 120.5) and WT (775.4 ± 72.64) mice in OF intermediate zone (t_{24} = -2.704, p = .012). **(G)** Distance

travelled (m) by *Pdzd8^{tm1b}* (71.12 ± 5.132) and WT (51.12 ± 5.718) mice in OF intermediate zone (t_{24} = -2.565, p = .017). **(H)** OF intermediate zone (800 cm^2). **(I)** No. entries by *Pdzd8^{tm1b}* (415.0 ± 23.89) and WT (372.1 ± 37.69) mice to OF outer zone (t_{24} = 0.925, p = .364). **(J)** Time (s) spent by *Pdzd8^{tm1b}* (2340.0 ± 137.2) and WT (2743.0 ± 80.83) mice in OF outer zone (t_{24} = 2.619, p = .015). **(K)** Distance travelled (m) by *Pdzd8^{tm1b}* (97.25 ± 13.22) and WT (89.23 ± 5.938) mice in OF outer zone (U = 73, p = .595). **(L)** OF outer zone (700 cm^2). **(M)** No. jumps by *Pdzd8^{tm1b}* (n = 11) and WT (n = 12) mice in 10-min intervals in OF. *Pdzd8^{tm1b}* mice increased their jumping over time (Friedman's ANOVA: χ^2_5 = 13.272, p = .021) but WT mice did not (Friedman's ANOVA: χ^2_5 = 3.795, p = .579). **(N)** No. wall supported rears by *Pdzd8^{tm1b}* (166.8 ± 23.43) and WT (231.3 ± 24.23) mice in OF (t_{21} = 1.908, p = .0702). **(O)** No. unsupported rears by *Pdzd8^{tm1b}* (331.5 ± 39.0) and WT (251.1 ± 35.12) mice in OF (t_{21} = 1.536, p = .1395). (**P**) Time (s) spent self-grooming by *Pdzd8^{tm1b}* (333.7 ± 61.64) and WT (348.6 ± 60.85) mice in OF (independent *t*-test: t_{21} = 0.1710, p = .8659). Data are plotted as mean ± SEM. *p < .05; **p < .01 vs. WT.

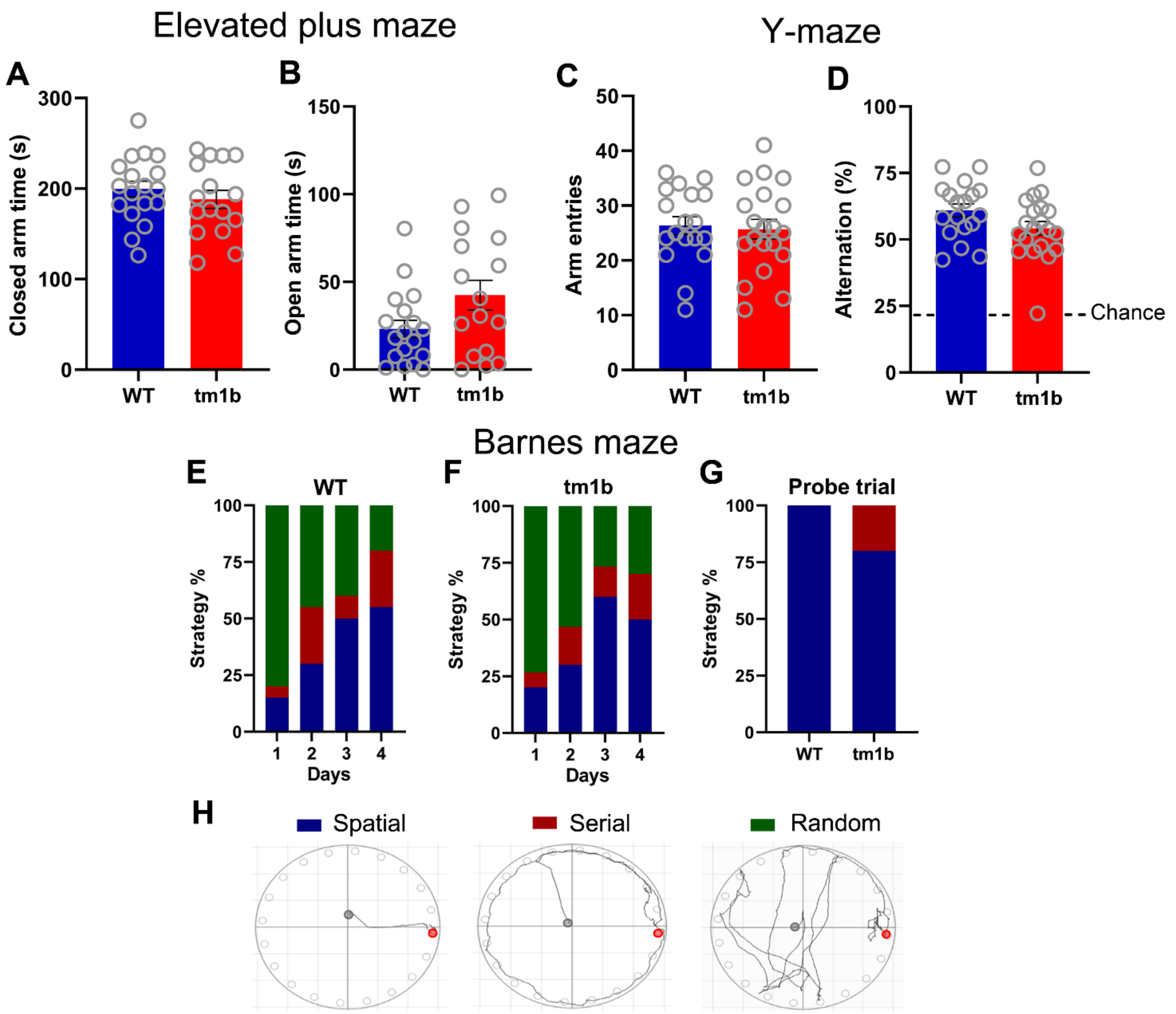

Figure S11. Behavioral parameters of *Pdzd8*^{tm1b} mice in elevated plus maze (EPM), Y-maze and Barnes maze. **(A)** Time (s) spent by *Pdzd8*^{tm1b} (n = 16; 42.4 ± 8.497) and WT (n = 18; 23.21 ± 5.021) mice on open arms of EPM (t_{32} = 1.997, p = .0544). **(B)** Time (s) spent by *Pdzd8*^{tm1b} (188.1 ± 10.02) and WT (199.6 ± 8.643) mice in closed arms of EPM (t_{32} = 0.874, p = .388). **(C)** No. arm entries by *Pdzd8*^{tm1b} (n = 20; 25.7 ± 1.77) and WT (n = 18; 26.33 ± 1.635) mice in Y-maze (independent t-test: t_{36} = 0.2610, p = .7956). **(D)** Spontaneous alternation (%) by *Pdzd8*^{tm1b} (54.24 ± 2.589) and WT (60.92 ± 2.47) mice in Y-maze (independent t-test: t_{36} = 1.857, p = .0715. One-sample t-test, *Pdzd8*^{tm1b} vs. chance (22%): t_{19} = 12.45, p < .0001; WT vs. chance: t_{17} = 15.76, p < .0001). Broken line indicates chance level. **(E)** Navigation strategies (%: random, serial, spatial) employed by WT mice (n = 10) during training in Barnes maze. WT mice showed a gradual increase in spatial approaches (Fisher's exact test: p = .006). **(F)** Navigation strategies (%: random, serial, spatial) employed by *Pdzd8*^{tm1b} mice (n = 15) during training in Barnes maze. *Pdzd8*^{tm1b} mice showed a gradual increase in spatial approaches (Fisher's exact test: p = .004). **(G)**

Navigation strategies (%: random, serial, spatial) employed by *Pdzd8*^{tm1b} and WT mice during probe trial in Barnes maze (Fisher's exact test: p = .25). **(H)** Representative track plots depicting different navigation strategies employed in the Barnes maze. Data are plotted as mean ± SEM.

SUPPLEMENTAL VIDEOS

Video S1

Provided as a separate file (Video S1.mp4). Spontaneous repetitive hindlimb jumping by group housed *Pdzd8*^{tm1b} mouse in the home cage (length: 19 s).

Video S2

Provided as a separate file (Video S2.mp4). Spontaneous repetitive hindlimb jumping by singly housed *Pdzd8*^{tm1b} mouse in the home cage (length: 13 s).

SUPPLEMENTAL EXPERIMENTAL PROCEDURES

Ethical Approvals

The human study was approved by the Sultan Qaboos University Ethical Committee. Informed consent was obtained from the parents of the affected individuals using a process that adhered to the tenets of the Declaration of Helsinki. Ethnically matched Omani controls were recruited from members of the Leeds Omani Society, using a process approved by the Leeds Central Research Ethics Committee (reference number 08/H1313/17). The mouse study was conducted in accordance with the UK Animals (Scientific Procedures) Act 1986 under UK Home Office licences and approved by Animal Welfare and Ethical Review Bodies at the University of Leeds, University of Bristol, and Wellcome Trust Sanger Institute (WTSI).

Sequencing and Variant Identification

Peripheral blood samples were withdrawn from each individual by venipunture at the cubital fossa for genomic DNA extraction using a QIAamp DNA Blood Mini Kit (Qiagen, Manchester, UK). Saliva was collected from controls into Oragene tubes (DNA Genotek, Ontario, Canada) and genomic DNA extracted using the manufacturer's protocol.

Homozygosity mapping and whole-exome sequencing (WES) were conducted according to protocols adapted from methods previously described (5). WES libraries were prepared from genomic DNA using the SureSelect Target Enrichment v4 reagent (Agilent Technologies, Wokingham, UK). These were paired end sequenced on a HiSeq 2500 System (Illumina, Little Chesterford, UK). Reads were aligned to the GRCh37 (hg19) reference human genome using Bowtie2. Picard and the Genome Analysis Toolkit were used to process alignments in the SAM/BAM format. Variants were annotated with ANNOVAR (6), then synonymous variants, intronic variants beyond the splice donor and acceptor sites, variants with a minor allele frequency >1% in the Genome Aggregation Database (gnomAD) (http://gnomad.broadinstitute.org/) and those with a read depth <10 were excluded. To check for homozygous regions, sequence variants were analysed using AgileVariantMapper and presented as a multi-Ideogram using AgileMultiIdeogram software (http://www.dna-leeds.co.uk/autozygosity).

Variants were prioritized for further analysis based on presence in homozygous regions and Combined Annotation Dependent Depletion (CADD) scores (http://cadd.gs.washington.edu). Segregation in affected and unaffected relatives was determined by PCR and Sanger sequencing. PCR products were incubated with ExoSAP-IT (Applied Biosystems, Warrington, UK), cycle sequenced using BigDye Terminator v1.1

(Applied Biosystems) and size-fractionated through a 3130xl Genetic Analyzer (Applied Biosystems) with results analysed using Sequencing Analysis Software v5.2 (Applied Biosystems). The ANKRD2 WT protein and the p.R328W variant were modeled using the Phyre2 Protein Fold Recognition Server (7).

Bulk Human Brain Gene Expression Analysis

The Allen Human Brain Atlas publishes a rich dataset of gene expression across brain regions, from age 8 wpc to adult ages (2). BrainSpan data analysis of *PDZD8* and the *CYC1* reference gene (3) was performed. RNA-seq expression measured in RPKM was obtained from the BrainSpan project data and summarized to Gencode v10 exons for selected annotated brain tissues aged 8 wpc to 23 years. To obtain a moving average across ages, a polynomial was fitted to the data.

Drosophila melanogaster

Fly Stocks and Husbandry

Fruit flies (*D. melanogaster*) were raised in a 25°C humidified room, with a 12:12 light:dark cycle. Flies were maintained in plastic vials containing 7 ml sugar-yeast-agar medium (8). Larvae were raised 100 per vial and supplemented with live yeast. Upon eclosion, flies were separated using ice anaesthesia into single-sex groups of ten, and the females supplemented with live yeast. The UAS-*CG10362*-RNAi (v8317) line (9) (hereafter referred to as UAS-RNAi) was obtained from the Vienna Drosophila Resource Center (VDRC, www.vdrc.at); the *Act5C*-Gal4 ubiquitous driver line was donated by Tracey Chapman (University of East Anglia, UK); and the Canton-Special (CS) strain, used as a WT control, was donated by Thomas Price (University of Liverpool, UK). The UAS-RNAi and *Act5C*-Gal4 lines were backcrossed to CS for five generations (average 96.88% CS genome at N_5). Flies were collected under ice anaesthesia and allowed to recover prior to phenotypic analysis; only males were assessed because *CG10362* is located on the X chromosome.

CG10362 Knockdown

To inhibit expression of *CG10362*, UAS-Gal4-mediated RNA interference (RNAi) was used (10). The UAS-RNAi line was crossed to *Act5C*-Gal4 (both lines CS N_5) to induce ubiquitous expression of a specific 326-bp hairpin structure (*CG10362*-RNAi) that inhibits expression of the target *CG10362* gene (*PDZD8* ortholog) via RNAi. To confirm knockdown (KD) of *CG10362*, whole adult (4–8-day-old) *Act5C*-Gal4 x UAS-RNAi F_1 (*CG10362*-KD), UAS-*CG10362*-RNAi and *Act5C*-Gal4 males were snap frozen in liquid N_2. RNA was extracted

from four pools of 8–10 male flies per line using a Direct-zol RNA MiniPrep Kit (Zymo Research, Irvine, USA). cDNA was synthesized from the RNA using the SuperScript First-Strand Synthesis System (Invitrogen). *eEF1α1* (*CG8280*) was selected as a stably expressed internal reference gene for data normalization (11).

To determine the relative expression of *CG10632*, a SYBR Green Quantitative RT-qPCR Kit (Sigma-Aldrich, Gillingham, UK) was used with forward (5'-GAC CTT CTG CTG GAC ATC AAC-3') and reverse (5'-CAG TGC GTG TAG GGC TTT CT-3') primers to amplify *CG10362*, and with forward (5'-GTC TGG AGG CAA TGT GCT TT-3') and reverse (5'-AAT ATG ATG TCG CCC TGG TT-3') primers to amplify *eEF1α1*. All primers were designed with a melting temperature of 60 ± 1°C and a CG content of 20–80%. Primer pairs were tested for efficiency using a 10x dilution series. Quantification of *CG10632* transcript levels relative to the *eEF1α1* reference gene was performed using the Pfaffl method (12). Ct values for samples were quantified against the lowest value across five biological replicates, using CFX Manager software (Bio-Rad, Watford, UK).

Aversive Olfactory Conditioning

Two odours, 4-methylcyclohexanol (MCH) and 3-octanol (OCT) diluted in light mineral oil (Sigma-Aldrich), were used as odorants. Conditioning and memory tests were performed on samples of 50 adult flies. Each sample of flies was conditioned against MCH or OCT for 60 seconds by exposure to an odorant and simultaneous stimulation with mechanical shock (2-second pulses delivered every 5 seconds) (13). This was followed by an interval of 30 seconds with room air before exposure to the second odorant without shock for 60 seconds. After an interval of 30 seconds with room air, flies tested for a preference between the two odours over 2 minutes. Flies were counted as having learnt if they chose the odour that was not simultaneous with mechanical shock. To calculate a learning index, the number of flies that did not learn was subtracted from the number of flies that learnt, and then divided by the total number of flies tested. An independent cohort of 50 flies would then be trained against the opposite odorant combination and these learning indices averaged for one data point.

Courtship Conditioning

Courtship conditioning is a well-established assay of *Drosophila* associative learning and memory, in which male flies that have had their copulation attempts rejected by pre-mated non-receptive females respond by suppressing their subsequent courtship behaviour. This learned suppression of courtship behavior is measured over time, thereby serving as an

indicator of learning and memory (14, 15). After exposure to a pre-mated CS female over a training period (1 hour or 5 hours), the male was isolated for a period of time (30 minutes or 48 hours) before being exposed to a virgin CS female for 10 minutes, during which the proportion of time the male performed stereotypic courtship behaviors was measured (courtship index). To establish whether each trained male had learnt, the courtship index was compared with that of untrained males of the same genotype with no prior exposure to pre-mated females. A memory index was calculated by dividing the courtship index of each trained male by the average courtship index of all untrained males of the same genotype. This creates a scale whereby 0 represents total suppression, and 1 represents no suppression (16).

To assess short-term (30-minute) memory, males were conditioning by being placed individually into a courtship chamber (0.3 cm^3) with a pre-mated female for 1 hour. Males were then removed and isolated in food vials for 30 minutes before being exposed to a virgin female in another, fresh, courtship chamber. Pairs were videoed for 10 minutes or until successful copulation to establish courtship indices.

To assess long-term (48-hour) memory, males were conditioned by exposure to a pre-mated female in a courtship chamber (8 cm^3) containing food for 5 hours (13). Males were then isolated in food vials for 0 hours (learning) or 48 hours before being tested via exposure to a virgin female in a courtship chamber (1.4 cm^3) for 10 minutes, where the courtship index was calculated.

Mice

The murine *Pdzd8* gene comprises five exons that encode a single transcript (NM_001033222). C57BL/6NTac-*Pdzd8*$^{\mathrm{tm1b(EUCOMM)Wtsi}}$ mice were generated in the European Conditional Mouse Mutagenesis (EUCOMM) program at WTSI (Hinxton, UK) by replacing an 835-bp sequence including exon 3 (103 bp) with a *lacZ* expression cassette (Figure S1A). This created a frameshift that changed the phenylalanine (TTC) and isoleucine (ATT) at positions 333 and 334 to an asparagine (AAT) and a termination codon (TAA) (p.F333Nfs1*) (Figure S1C) (17). Heterozygotes were intercrossed to generate *Pdzd8*^{tm1b}, heterozygous and WT littermates for phenotypic testing. Pups were weaned at 4 weeks of age and grouped housed (3–5 mice/cage) with same-sex littermates under a 12 hour light/dark cycle (lights on at 06.00). Pelleted feed (CRM-P, SDS Diets, Braintree, UK) and water were provided *ad libitum*. DNA was extracted from ear biopsies taken at weaning. Mice were genotyped by multiplex PCR with primers annealing to the deleted and introduced sequences in the reporter-tagged deletion allele (Figure S1B, C) using the thermocycling

program of: 95°C for 3 min, followed by 30 cycles of 94°C for 30 s, 53°C for 60 s, 72°C for 40 s, followed by 72°C for 10 min. PCR products were visualized using agarose gel electrophoresis, with a 554-bp band indicating the *Pdzd8*^{tm1b} allele, and a 488-bp band indicating the WT allele.

Antibodies

Rabbit polyclonal antibody PA5-46771 to PDZD8 (1:500; overnight) (Invitrogen), with a 50-aa epitope (935–984 aa) between the C-terminal C1 and CC domains, was used for western blotting. Mouse monoclonal antibody A1978 to β-actin (1:5,000; 1 hour) (Sigma-Aldrich, Gillingham, UK) was used as a loading control.

Real-time qRT-PCR of *Pdzd8* transcript

Mice were killed by cervical dislocation and their brains were quickly extracted and snap frozen in liquid N_2. RNA was extracted from whole brain using an Ambion RNAqueous Total RNA Isolation Kit (Life Technologies, Paisley, UK), followed by spectrophotometric analysis of purity/integrity by 260 nm analysis of RNA concentration and 260/280 nm ratio analysis of RNA purity. One µg of each RNA sample was converted into cDNA using SuperScript III Reverse Transcriptase (Life Technologies). The cDNA was stored at −80°C before analysis by qRT-PCR using a QuantStudio 3 Real-Time PCR System (Life Technologies) with SYBR Green PCR Master Mix (Life Technologies) and the following primers: *Pdzd8*: forward (5'-TTG AGC TGA GCA GTG GTG TT-3') in exon 3 and reverse (5'-AGA CGA AGT GTA AGT CCG ACA-3') in exon 4; *lacZ*: forward (5'-ATG GGT AAC AGT CTT GGC GG-3') and reverse (5'-AAT CAG CGA CTG ATC CAC CC-3'); *Hprt*: forward (5'-TGC TGA CCT GCT GGA TTA CAT-3') and reverse (5'-TTT ATG TCC CCC GTT GAC TGA T-3'); *B2m*: forward (5'-CTG GTG CTT GTC TCA CTG ACC-3') and reverse (5'-CGT AGC AGT TCA GTA TGT TCG G -3'). Data were normalized to the *Hprt* and *B2m* reference genes. Analysis was carried out using the $2^{-\Delta\Delta Ct}$ method (4).

Mouse Behavioral Testing

All behavioral experiments were conducted using early adults over 8 weeks of age. Mice were handled for one week prior to behavioral testing. Mice were transferred to the experimental room 30 mins prior to the start of testing. All apparatus was cleaned with 70% ethanol between each mouse. All experiments were conducted between 9am and 5pm. A webcam was positioned directly above the apparatus to record the trials using ANY-maze

Video Tracking Software (Stoelting, Dublin, Ireland). Experimenters were blinded to genotype during behavioral testing.

Open Field

The open field test was conducted according to a protocol adapted from a previously described method (18). The open field arena had semi-transparent (frosted) Perspex walls, with an internal diameter of 40 x 40 cm (1,600 cm^2). The floor of the arena was white plastic. A circular white outer wall (30 cm away from the arena wall) prevented the testing room from being visible during the trial. Lighting of the maze was measured using a luminance meter at ~200 lux at the center of the arena.

Each mouse was placed into the arena facing the same wall and left to explore undisturbed for 60 minutes. The tracking software divided the arena into the following zones, using the following dimensions: outer zone, 5 cm from the outer wall (700 cm^2, 43.75% total area); inner zone (100 cm^2; 6.25% total area); intermediate zone (800 cm^2; 50% total area). Time spent in and entries to each of the zones and distance travelled were measured. Entry into a zone was recorded when the centre point of the animal crossed into the specific zone. Rearing, jumping and grooming were recorded during the trial by the experimenter.

Elevated Plus Maze

The elevated plus maze test was conducted according to a protocol adapted from a previously described method (18). The elevated plus maze consisted of two closed arms with white opaque walls and two open arms made of matt white acrylic. All arms measured 30 cm long and 5 cm wide. There was a central square of 25 cm^2 where all arms met. The mouse was placed into the plus maze in the central square, facing the opposing open arm from the experimenter, and subsequently allowed to explore the maze for 5 mins. Time in and entries made to the open, closed and central square were measured, in addition to the number of head dips over the side of the open arms. An entry into an arm or central square was recorded when the center point of the animal crossed into the specific zone.

Y-maze

The Y-Maze spontaneous alternation test was conducted according to a protocol adapted from a previously described method (19). The Y-maze was constructed from three identical arms (35 x 5 x 15 cm) made of matt white acrylic, placed at an angle of 120° from each other. Mice were placed into the end of one of the arms (start arm was counterbalanced between genotype and sex) and allowed to roam freely through the maze for 5 minutes. The

number of arm entries, including possible returns to the same arm, was recorded and spontaneous alternations were scored offline later by an experimenter blind to genotype and sex. Entry into an arm was defined as when all four paws of the mouse had entered the arm. An alternation occurs when a mouse enters three different arms consecutively. The experimenter calculated the alternation percentage by dividing the number of alternations by the total number of arm entries minus 2. Thus, if a mouse made the following arm entries (C, B, A, B, C, B, A, C), the total number of entries minus 2 would be 6 and the total number of alternations would be 4 (CBA, ABC, CBA, BAC), with an alternation percentage of 67%. Mice could repeat entries into a single arm in this variant of the Y-maze procedure, resulting in a chance performance level of 22% alternation (2/9), relative to the 50% alternation chance performance level in versions of this task that preclude multiple entries into the same arm.

Barnes Maze

The Barnes maze test was conducted according to a protocol adapted from a previously described method (20). The maze consisted of a 9mm thick satin white PVC circular arena (122 cm diameter) with 20 equidistant holes (5 cm diameter) around the perimeter (7.5 cm from the edge). The maze had no perimeter walls and was raised 40 cm from the ground. The maze was surrounded by various distal spatial cues. Overhead room lights and two 60w lamps illuminated the maze to provide an aversive stimulus to escape. An escape box (25 x 6 x 5 cm) was attached to the underside of the maze, allowing escape from the arena via one of the 20 holes (the target hole).

Mice initially received a 2-minute habituation session to the maze and escape box. The acquisition phase consisted of 8 trials over 4 days (2 trials/day). The maximum duration of each trial was 120 s with a 2-hr inter-trial interval. For each mouse, the escape box location (either the east or west hole, counterbalanced between genotypes) remained constant throughout acquisition. Immediately preceding each trial, the mouse was placed onto the center of the arena enclosed in a cardboard chamber. Once the chamber was removed, the mouse had free access to the entire arena for 120 s. If the mouse located and entered the escape box within the 120 s, it was allowed to spent a 60 s inside before being returned to the home cage. If the mouse failed to enter the escape box within 120 s, a 5L glass beaker was placed over the mouse and used to guide it to the target hole. The beaker was left over the target hole for 60 s; if the mouse failed to enter in this period, it was gently guided into the hole using the edge of the beaker.

During acquisition trials, the following parameters were used to assess spatial learning: latency (s) to enter the target hole; latency (s) of the mouse's head to enter the target hole for the first time; and distance travelled (m) before the mouse made its first head entry into the target hole (primary path length).

Twenty-four hours after the last acquisition day, a probe trial was conducted, during which the escape box was removed and the mouse was allowed to explore the arena for 80 s. Three measures were used to assess spatial memory during the probe trial: time spent in the target quadrant (a geometric area covering 25% of the arena with the escape hole in the center of five holes); time spent in the target sector (a geometric area covering 5% of the arena for each of the 20 holes); and time spent in and number of entries to the target hole annulus (a 7 cm diameter circle centered on the target hole).

Search strategies in the Barnes maze were classified using the BUNS (Barnes-maze unbiased strategy) algorithm (21). Fisher's exact test was used to analyse the spatial strategy data.

Statistical Analysis

Statistical analysis was performed using SPSS and GraphPad Prism. Data were tested for normality using the Shapiro-Wilk test, and for and homoscedasticity using Levene's test. We used Student's *t* tests (with Welch corrections for heteroscedasticity), and two-way repeated-measures analysis of variance (ANOVA) when data passed normality and homoscedasticity assumptions. If applicable, the Greenhouse–Geisser adjustment was used to correct for violations of sphericity. Significant ANOVA interactions were analysed further using simple main effect analysis with Fisher's least significant difference (LSD). If the data violated normality, it was square root transformed (SQRT) or Mann-Whitney tests and Friedman's ANOVA were used. Unless otherwise stated, α was set at .05. Graphs were prepared using GraphPad Prism version 6.

Electrophysiology

Slice Preparation

Transverse hippocampal slices were prepared from 4-6-week-old male and female C57BL/6NTac, WT or *Pdzd8*^{tm1b} mice. Brains were immediately removed following cervical dislocation and immersed in ice-cold cutting artificial cerebral spinal fluid (aCSF) containing (in mM): 205 sucrose, 10 glucose, 26 $NaHCO_3$, 2.5 KCl, 1.25 NaH_2PO_4, 0.5 $CaCl_2$, and 5 $MgSO_4$. Individual hippocampi were mounted on agar and 400 µm thick slices cut using a microslicer (VT1200S, Leica Microsystems). Following preparation, slices were transferred

to aCSF containing (in mM): 119 NaCl, 10 D-glucose, 26 $NaHCO_3$, 2.5 KCl, 1 NaH_2PO_4, 1.3 $MgSO_4$, and 2.5 $CaCl_2$, maintained at 35°C for 30 min and then stored at room temperature for a further 30 mins before recording. All solutions were saturated with 95% O_2 and 5% CO_2 and had osmolarity 300-310 mOsm.

Recording

Slices were placed in a submerged recording chamber perfused with aCSF at 33°C at 2-3 ml/min. Slices were visualized using IR-DIC optics. Extracellular recording electrodes with a resistance of 1-3 MΩ were pulled from borosilicate filamented glass capillaries and filled with aCSF. Field excitatory postsynaptic potentials (fEPSPs) were evoked using bipolar stimulating electrodes placed in stratum radiatum. All recordings were made using a MultiClamp 700B amplifier (Molecular Devices, Wokingham, UK) and were digitized at 10 kHz and filtered at 3 kHz. Three different LTP induction protocols were employed. Theta burst stimulation (TBS) consisted of 10 bursts at 5 Hz where each burst consisted of 5 stimuli at 100 Hz. This was either applied once (1*TBS) or 3 times separated by 10s (3*TBS). High frequency stimulation (HFS) consisted of one burst of 100 stimuli at 100 Hz (1*HFS). All experiments within groups were interleaved and performed with experimenter blind to the animal genotype.

Data Analysis

All data are expressed as mean ± SEM. Example traces shown are averages of 10-20 consecutive sweeps. Statistical significance was assessed using paired or unpaired Student's *t*-tests as appropriate and the level of significance set at $p < .05$. Synaptic strength for extracellular recordings was measured as the initial slope (20-50%) of the field potential response.

Structural MRI

Adult mice (16 weeks old) were terminally anesthetized and intracardially perfused with 30 ml of 0.1 M PBS containing 10 U/ml heparin and 2 mM gadoterate meglumine (Dotarem, Guerbet, Solihull, UK), followed by 30 ml of 4% paraformaldehyde (PFA) containing 2 mM gadoterate meglumine. Perfusions were performed at a rate of approximately 1.0 ml/min. After perfusion, the brain and remaining skull structures were incubated in 4% PFA + 2 mM gadoterate meglumine overnight at 4°C and transferred to 0.1 M PBS containing 2 mM gadoterate meglumine and 0.02% sodium azide for at least 1 month prior to MRI scanning.

A multi-channel 7.0 Tesla MRI scanner (Agilent Technologies, Palo Alto, USA) was used to image the brains within their skulls. Sixteen custom-built solenoid coils were used to image the brains in parallel. The parameters for the MRI scan were as follows: T2-weighted 3D fast spin-echo sequence, with a cylindrical acquisition of k-space, and with a TR of 350 ms, and TEs of 12 ms per echo for 6 echoes, 2 averages, field-of-view of 20 x 20 x 25 mm^3 and matrix size = 504 x 504 x 630 giving an image with 0.040 mm isotropic voxels. The total scanning time was ~14 hr.

To visualize and compare any changes in the mouse brains, the images were linearly (6 parameter followed by a 12 parameter) and non-linearly registered together. At completion of this registration, all scans were iteratively linearly and non-linearly aligned to each other to create a population atlas representing the average anatomy of the entire study sample. All scans were thus deformed into alignment with each other to a consensus average in an unbiased fashion. This allows for analysis of the deformations required to register the anatomy of each individual mouse into the final atlas space.

Volumetric changes were calculated on a regional and a voxel-wise basis. The Jacobian determinants of the deformation fields were computed and analyzed to measure the volume differences between subjects at every voxel. To compute the volume of brain regions in all the input images, a pre-existing classified MRI atlas encompassing 159 different segmented structures throughout the brain was warped onto the population atlas. To assess differences in specific brain regions, we normalized the volume of each region to the absolute brain volume, using the formula `[individual absolute volume region / individual absolute volume whole brain * mean absolute volume whole brain]`, and reported the relative volume as % total brain volume. A linear model with genotype and sex as predictors was fitted to the absolute and relative volume of every region independently and to every voxel independently (voxel-wise statistics) in the brains of *Pdzd8*^{tm1b} and WT mice, with a false discovery rate (FDR) threshold of 1%. Multiple comparisons were controlled for using the FDR within the RMINC package for R.

SUPPLEMENTAL REFERENCES

1. Bakken TE, Jorstad NL, Hu Q, Lake BB, Tian W, Kalmbach BE, *et al*. (2021): Comparative cellular analysis of motor cortex in human, marmoset and mouse. *Nature* 598: 111–119.
2. Miller JA, Ding SL, Sunkin SM, Smith KA, Ng L, Szafer A, *et al*. (2014): Transcriptional landscape of the prenatal human brain. *Nature* 508: 199–206.
3. Penna I, Vella S, Gigoni A, Russo C, Cancedda R, Pagano A. (2011): Selection of candidate housekeeping genes for normalization in human postmortem brain samples. *Int J Mol Sci* 12: 5461–5470.
4. Livak KJ, Schmittgen TD (2001): Analysis of relative gene expression data using real-time quantitative PCR and the 2(-Delta Delta C(T)) Method. *Methods* 25: 402–408.
5. Al-Amri A, Al Saegh A, Al-Mamari W, El-Asrag ME, Ivorra JL, Cardno AG, *et al*. (2016): Homozygous single base deletion in TUSC3 causes intellectual disability with developmental delay in an Omani family. *Am J Med Genet A* 170: 1826–1831.
6. Wang K, Li M, Hakonarson H (2010): ANNOVAR: functional annotation of genetic variants from high-throughput sequencing data. *Nucleic Acids Res* 38: e164.
7. Kelley LA, Mezulis S, Yates CM, Wass MN, Sternberg MJ (2015): The Phyre2 web portal for protein modeling, prediction and analysis. *Nat Protoc* 10: 845–858.
8. Bass TM, Grandison RC, Wong R, Martinez P, Partridge L, Piper MD (2007): Optimization of dietary restriction protocols in Drosophila. *J Gerontol A Biol Sci Med Sci* 62: 1071–1081.
9. Dietzl G, Chen D, Schnorrer F, Su KC, Barinova Y, Fellner M, *et al*. (2007): A genome-wide transgenic RNAi library for conditional gene inactivation in Drosophila. *Nature* 448: 151–156.
10. Brand AH, Perrimon N (1993): Targeted gene expression as a means of altering cell fates and generating dominant phenotypes. *Development* 118: 401–415.
11. Ling D, Salvaterra PM (2011): Robust RT-qPCR data normalization: validation and selection of internal reference genes during post-experimental data analysis. *PLoS One* 6: e17762.
12. Pfaffl MW (2001): A new mathematical model for relative quantification in real-time RT-PCR. *Nucleic Acids Res* 29: e45.
13. Mery F, Kawecki TJ (2005): A cost of long-term memory in Drosophila. *Science* 308: 1148.

14. McBride SM, Giuliani G, Choi C, Krause P, Correale D, Watson K, *et al*. (1999): Mushroom body ablation impairs short-term memory and long-term memory of courtship conditioning in Drosophila melanogaster. *Neuron* 24: 967–977.
15. Koemans TS, Oppitz C, Donders RAT, van Bokhoven H, Schenck A, Keleman K, *et al*. (2017): Drosophila courtship conditioning as a measure of learning and memory. *J Vis Exp* 124: e55808.
16. Ejima A, Griffith LC (2011): Assay for courtship suppression in Drosophila. *Cold Spring Harb Protoc* 2011: pdb.prot5575.
17. Friedel RH, Seisenberger C, Kaloff C, Wurst W (2007): EUCOMM--the European conditional mouse mutagenesis program. *Brief Funct Genomic Proteomic* 6: 180–185.
18. Dachtler J, Glasper J, Cohen RN, Ivorra JL, Swiffen DJ, Jackson AJ, *et al*. (2014): Deletion of α-neurexin II results in autism-related behaviors in mice. *Transl Psychiatry* 4: e484.
19. Holcomb LA, Gordon MN, Jantzen P, Hsiao K, Duff K, Morgan D (1999): Behavioral changes in transgenic mice expressing both amyloid precursor protein and presenilin-1 mutations: lack of association with amyloid deposits. *Behav Genet* 29: 177–185.
20. Riedel G, Robinson L, Crouch B (2018): Spatial learning and flexibility in 129S2/SvHsd and C57BL/6J mouse strains using different variants of the Barnes maze. *Behav Pharmacol* 29: 688–700.
21. Illouz T, Madar R, Clague C, Griffioen KJ, Louzoun Y, Okun E (2016): Unbiased classification of spatial strategies in the Barnes maze. *Bioinformatics* 32: 3314–3320.
